# A general theory of polymer ejection tested in a quasi two-dimensional space

**DOI:** 10.1038/s41598-021-94054-2

**Published:** 2021-07-19

**Authors:** Pai-Yi Hsiao, Wei-Yei Chen

**Affiliations:** 1 Department of Engineering and System Science, National Tsing Hua University, Hsinchu, Taiwan, Republic of China; 2 Institute of Nuclear Engineering and Science, National Tsing Hua University, Hsinchu, Taiwan, Republic of China

**Keywords:** Biophysics, Biological physics, Molecular engineering, Nanopores

## Abstract

A general ejection theory of polymer is developed in a two- and three-dimensional space. A polymer is confined initially in a cavity and ejects spontaneously to the outer space through a nanopore channel without the help of any external stimulus. A reflective wall boundary is set at the pore entrance to prevent the falling of the head monomer of chain into the cavity. Three stages are distinguished in a process: (1) an entering stage, in which the head monomer enters the pore to search for a way to traverse the pore channel, (2) a main ejection stage, in which the chain body is transported from the cavity to the outer space, (3) a leaving stage, in which the tail monomer passes through and leaves the pore channel. Depending on the number of the monomers remaining in the cavity, the main ejection stage can be divided into the confined and the non-confined stages. The non-confined stage can be further split into the thermal escape and the entropic pulling stages. The Onsager’s variational principle is applied to derive the kinetics equation of ejection. The escape time is calculated from the corresponding Kramers’ escape problem. Extensive molecular dynamics simulations are then performed in a quasi two-dimensional space to verify the theory. The variation of the ejection speed is carefully examined. The decreasing behavior of the number of monomers in the cavity is studied in details. The scaling properties of the spending time at each processing stage are investigated systematically by varying the chain length, the cavity diameter, and the initial volume fraction of chain. The results of simulation support firmly the predictions of the theory, cross-checked in the studies of various topics. In combining with the previous investigations in the three-dimensional space, the generalized theory is very robust, able to explain the two seemly different phenomena, polymer ejection and polymer translocation, together under the same theoretical framework in the two space dimensions.

## Introduction

One of the main objectives in bionanotechnology is to develop efficient techniques for control or manipulation of biomacromolecules for diverse applications^1–3^. Recent advances have demonstrated the capabilities to imprison or encapsulate single DNA or RNA chains in a small trap or cavity for preserving or transportation^4–7^. The molecular chains are released at the moment of necessary to a destination space for sensing or therapeutic purposes^8,9^ In Nature, bacteriophage is a virus mastering the skill^10,11^. It infects bacteria by ejecting the genetic materials encapsulated in the viral capsid through a small channel into the host cell for replication. The ejecting process occurs spontaneously where no external driving force intervenes in pressing or pulling the chain out of the cavity. It is a result of the favor of thermodynamics because the chemical potential inside the capsid is higher than the outside^12,13^.

The ejecting problem takes place at nanometer scales where the thermal fluctuations have great influences. The stochastic nature of the surroundings blurs the deterministic trajectory of ejection issued from the confinement. It renders the classical method, by solving the problem under the framework of continuum mechanics, insufficient in describing the properties of such a process^12–20^. External factors such as temperature^21^, acidity of environment^22^, ions and salinity^23^, osmotic pressure^20,24^, *etc.*, can disturb the progress of ejection^25^. Other effects like protein binding in the cytoplasm of host cell^18,21^ and condensation of the ejected chain^26^ can help the ejection. The factors and effects complicate the process and make the system difficult to be predicted.

A fundamental approach to study the ejection problem at a primitive level starts with the calculation of the change of the free energy^27–32^. The ejection of chain results in a decrease of the free energy, which drives the chain out of the cavity. Polymer physics have provided a sophisticated way to estimate chain free energy under diverse situations via scaling analysis^33–35^. By adopting the free energy of polymer in a semidilute solution, Muthukumar^36^ predicted that the ejection should be $\tau _{\mathrm{ej}}\sim N^{1+(1/3\nu )}\phi _0^{-1/3\nu }$ with $\nu$ being the Flory exponent, where the initial volume fraction of chain in the cavity is $\phi _0$ and the chain length is *N*. Cacciuto and Luijten^37^ reported later in the Monte Carlo simulations that $\tau _{\mathrm{ej}}$ should be $N^{1+\nu }\phi _0^{-1/(3\nu -1)}$. Sakaue and Yoshinaga^38^ made a careful study and pointed out an important fact that the volume fraction of the number of the monomers in the cavity decreases and thus, the driving chemical potential difference is not constant but decreases with time. They balanced the rate of the free energy change with the rate of the energy dissipation taking place in the solution near the pore within a correlation length $\xi$; an ejection time $\tau _{\mathrm{ej}}\sim N^{(2+\nu )/(3\nu )}\phi _0^{-(2+\nu )/(3\nu )}$ was predicted. They also envisaged that the ejection process was ended by a diffusion-like escape with the spending time $\tau _{\mathrm{D}}\sim D^2/{\mathfrak {D}}_{N_0}$ where *D* is the cavity diameter and ${\mathfrak {D}}_{N_0}$ the diffusion coefficient to diffuse the last segments of chain out of the cavity.

Recently, we have modified and extended the theory^39,40^. The new theory balanced the rate of the free energy change with the rate of the energy dissipation at the pore with a fraction coefficient varying during the process. Instead of treating the final escape in a diffusive way, we dealt with it as a formal translocation, with the free energy being the sum of the free energies from the two chain segments instantaneously expanding on the two sides of the pore. The ejection time $\tau _{\mathrm{ej}}\sim N^{(2+\nu )/(3\nu )}\phi _0^{-2/(3\nu )}$ was obtained in our theory. The results have been demonstrated correct by extensive molecular dynamics simulations. A double verification has been done under the *D*-fixed condition, which revealed a $N^{1/3}$-dependence for $\tau _{\mathrm{ej}}$, following our prediction. Ref.^38^, in contrast, expected a null dependence on *N*. Because the translocation theory is involved in the derivation, our theory successfully connects the two seemly different problems together, polymer ejection and polymer translocation^35,41–43^. The theory is applicable in the entire (*N*, *D*, $\phi _0$) parameter space. Moreover, we pinpointed the existence of a nucleation-like stage occurred prior to the main ejection, which takes care of the traversing of the heading monomers across the pore channel. A theory has been developed by regarding it as a Kramers’ escape problem and explained it in analogy to the nucleation phenomena^40^.

Despite of the above achievements, there are still several issues to be clarified or resolved. For example, how does the free energy landscape look like in term of the ejection coordinate under different ejection conditions? A good knowledge of the free energy landscape enables a well understanding of the physical pictures behind an ejection phenomenon. In the theory, we have divided the main ejection into the confined and the non-confined stages, and assumed that it is the entropic pulling which drives the chain out of the cavity in the latter stage. However, it is not always the case. A situation can happen if the chain length is not long compared to the cavity diameter—the segment length in the cavity can be still longer than the one from the outside at the moment when the process enters to the non-confined stage, and therefore, the driving force is not entropic at that moment. How shall we fix the problem? Is this particular situation detectable in simulations and what is its significance? In a real world, the encapsulating cavity may not be spherical. Researchers may enclose a biopolymer in a quasi-two-dimensional cage and release it later though a pore or a canal for applications. It addresses an important question, how to generalize the current theory for a two-dimensional space. The generalization can be served as a test of the robustness of the theory.

In this paper, a general theory for polymer ejection in a *d*-dimensional space is given in "General ejection theory in a *d*-dimensional space" section. An extensive molecular dynamics simulation is then performed in a quasi two-dimensional space to verify the generalization of the theory where the modeling and settings are described in "Simulation model and settings" section. The results are reported in "Results" section. The studied topics include the variation of the ejection speed during a process ("Ejection speed" section), the decrease of the number of the monomers in a confining cavity ("Decrease of number of monomers in the cavity" section), and the scaling behaviors of the decomposed time as a function of *N*, *D*, and $\phi _0$ in each stage ("Processing time analysis" section). The discussions and conclusions are given in "Discussions and conclusions" section.

## General ejection theory in a *d*-dimensional space

Consider the problem of a polymer ejecting in a *d*-dimensional space. The polymer is initially confined in a circular cavity and ejects through a pore channel into a semi-space. The cavity is a disk if $d=2$ and a sphere if $d=3$. The chain comprises *N* monomers, each has a diameter $\sigma$. The monomers are linearly connected by chemical bonds of length $\sigma$ too. The circular cavity has a diameter *D* and the pore length is $\ell _{\mathrm{p}}$. The pore diameter $d_{\mathrm{p}}$ is assumed small, which allows only one monomer to pass by the cross-section at a time. Under this setting, the initial volume fraction of monomers in the cavity is $\phi _0=N(\sigma /D)^{d}$. The head monomer is positioned at the entrance of the pore for starting. We assume that some mechanism exists to prevent falling of the head monomer into the interior of the cavity. Therefore, the ejection theory presented here does not include the searching of the pore entrance for the head monomer from the interior.

Extending the results of the previous study^39,40^, we generalize the scaling theory. An ejection process of polymer can be divided into three stages, namely the entering stage, the main ejection stage, and the leaving stage. The process can be described by two dependent state variables: *m*, denoting the number of monomers remaining in the cavity, and *s*, denoting the one having arrived in the semi-space. Because the pore channel allows accommodation of $m_{\mathrm{p}}$ monomers, where $m_{\mathrm{p}}=\ell _{\mathrm{p}}/\sigma$, we have $N=m+m_{\mathrm{p}}+s$. The negative *s* state ($-m_{\mathrm{p}}\le s \le 0$) occurs at the beginning, the “entering stage”, when the heading monomers strive to traverse the pore channel against the chemical potential difference $\Delta \mu _{\mathrm{cp}}$ created by the osmotic pressure between the cavity and the pore. An activation energy $E_{\mathrm{a}}$ is established, which is equal to $\Delta \mu _{\mathrm{cp}}$ multiplying $m_{\mathrm{p}}$. The rate of transition, also called the rate of escape, is described by an Arrhenius-type equation $\eta ^{-1}\exp \left( -E_{\mathrm{a}}/k_{{\mathrm{B}}}T\right)$ where $\eta$ is the friction coefficient^44,45^. A detail calculation given in Appendix A in the Supplementary Information (SI) predicts $\tau _{\mathrm{ent}}\sim \eta \exp \left( \frac{m_{\mathrm{p}}\Delta \mu _{\mathrm{cp}}}{k_{{\mathrm{B}}}T}\right)$ for $m_{\mathrm{p}}\Delta \mu _{\mathrm{cp}}\gg k_{{\mathrm{B}}}T$. If $\Delta \mu _{\mathrm{cp}}\ll 0$, we have $\tau _{\mathrm{ent}}\sim (\frac{\eta \sigma ^2}{|\Delta \mu _{\mathrm{cp}}|}) m_{\mathrm{p}}$. In between ($|\Delta \mu _{\mathrm{cp}}|$ is close 0), $\tau _{\mathrm{ent}}$ has an expression of the diffusion time $\frac{\eta \ell _{\mathrm{p}}^2}{2k_{{\mathrm{B}}}T}$.

The main ejection stage is started as the first monomer enters the semi-space, and ended as the last monomer leaves the cavity. In the language of the state variables, it happens when $0<s<N-m_{\mathrm{p}}$ or equivalently $N-m_{\mathrm{p}}>m>0$. The stage can be further subdivided into two stages according to the *m* value. At the confined stage, the chain feels the confining effect of the cavity and is “compressed” out of it through the pore. As *m* decreases and becomes eventually smaller than a critical number $N_*$, the remaining chain size is smaller than the cavity size. At that moment, there is no confining effect on the chain and the system is in the non-confined stage. The critical number $N_*$ has been shown to scale as $(D/\sigma )^{1/\nu }$ with $\nu =3/5$ in the three-dimensional space^40^. We will verify it later if the scaling is truly held by switching the Flory exponent to its two-dimensional value $\nu =3/(d+2)=3/4$.

The kinetics equation for the state variable *m* can be deduced from Onsager’s variational principle^40,46,47^ and reads as

1 $$\begin{aligned} \frac{\mathrm{d}m}{\mathrm{d}t}= -\frac{1}{\eta \sigma ^2}\frac{\mathrm{d}F}{\mathrm{d}m} \end{aligned}$$

where *F* is the free energy of the system. By using the blob theory, *F* can be shown to be $\sim k_{{\mathrm{B}}}T(m/N_*)^{z_1 +1}$ at the confined stage^34,48^. Here $z_1=1/(d\nu -1)$ is a key exponent appeared regularly in the des Cloizeaux theory for polymer solutions^49^ and we have expressed it in the general form for a *d*-dimensional space. The kinetics equation for *m* is thus

2 $$\begin{aligned} \frac{\mathrm{d}m}{\mathrm{d}t}\sim -\frac{z_1+1}{\Delta {\mathfrak {t}}} \left( \frac{m^{z_1}}{N_*^{z_1+1}}\right) \end{aligned}$$

where $\Delta {\mathfrak {t}}=\eta \sigma ^2/k_{{\mathrm{B}}}T$ is the characteristic time. It has been argued that $\eta$ exhibits a $N^{x_1}$ dependence with $x_1=1/3$ in the three-dimensional space^39,40^. A general formula for $x_1$ should be 1/*d*, which leads to $\Delta {\mathfrak {t}} \sim N^{1/d} \Delta {\mathfrak {t}}_0$. Here $\Delta {\mathfrak {t}}_0=\eta _{\mathrm{0}}\sigma ^2/k_{{\mathrm{B}}}T$ and $\eta _{\mathrm{0}}$ is the friction coefficient for a monomer. Intensive simulations will be performed later to verify it for the two-dimensional case.

At the non-confined stage, the free energy can be approximately described by

3 $$\begin{aligned} F \sim k_{{\mathrm{B}}}T\left[ (1-\gamma _{1})\ln (m+1) + (1-\gamma _{1})\ln (s+1) -(N-m_{\mathrm{p}})\ln q \right] \end{aligned}$$

where $\gamma _{1}$ is the entropic exponent and *q* is the effective coordination number. It is derived from $F \sim -k_{{\mathrm{B}}}T\ln (Z_{m}Z_{s})$ with $Z_{m}\sim (m+1)^{\gamma _{1}-1} q^{m}$ and $Z_{s}\sim (s+1)^{\gamma _{1}-1} q^{s}$ being the partition functions for a polymer of *m* and *s* monomers, respectively, tethered on a surface^35,50,51^. The value of $\gamma _{1}$ is about 0.687 for $d=3$^52^ and 0.955 for $d=2$^53^. To avoid the divergence of *F* at $m=0$ and $s=0$, we have remedied the expression by adding one to the base of the exponent $\gamma _{1}-1$ in the partition function to assure $Z_0=1$. The kinetics equation is thus obtained:

4 $$\begin{aligned} \frac{\mathrm{d}m}{\mathrm{d}t}\sim \frac{-1}{\Delta {\mathfrak {t}}} \left[ \frac{1-\gamma _{1}}{m+1} - \frac{1-\gamma _{1}}{s+1}\right] . \end{aligned}$$

This equation is valid only when $m<s$, or equivalently, $m<(N-m_{\mathrm{p}})/2$. It depicts the situation that the chain is pulled outward by the dominated entropic force from the outside. If *m* is much smaller than *s*, the second term on the right-hand side of the equation can be ignored and the kinetics exhibit a scaling variation like

5 $$\begin{aligned} \frac{\mathrm{d}m}{\mathrm{d}t}\sim \frac{-1}{\Delta {\mathfrak {t}}} m^{-1} \sim \frac{-1}{\Delta {\mathfrak {t}}_0} m^{-z_{\mathrm{2P}}}. \end{aligned}$$

It has been argued that the friction coefficient $\eta$ scales as $\eta _0 m^{y_{\mathrm{2P}}}$ with $y_{\mathrm{2P}}=2\nu -1$ because the rested *m* monomers in the cavity contribute to the energy dissipation at the non-confined stage^40^. As a result, the ejection speed shows $m^{-z_{\mathrm{2P}}}$ variation with $z_{\mathrm{2P}}=1+y_{\mathrm{2P}}=2\nu$.

Please notice that there exists an intermediate cavity size *D* with the corresponding $N_*$ larger than $(N-m_{\mathrm{p}})/2$ but smaller than $N-m_{\mathrm{p}}$. In this situation, the system enters to the non-confined stage with the exterior chain length being still shorter than the interior one. As a consequence, a free energy barrier has to be surmounted first. The escape theory is applied again and the required escape time can be shown to be $\tau _{\mathrm{2E}} \sim \frac{\eta _0\sigma ^2}{k_{{\mathrm{B}}}T}\left[ N^{2+x_1}I_{1}({\tilde{s}}_*) + N^{2+y_{\mathrm{2E}}}I_{\mathrm{2E}}({\tilde{s}}_*)\right]$ (cf. Appendix A in SI). After surmounting the free energy barrier, we have $s>m$ and the kinetics can be validly described by Eq. (5). The required time to pull the rest chain out of the cavity is $\tau _{\mathrm{2P}} \sim \Delta {\mathfrak {t}}_0 N_*^{1+z_{2P}}$.

The final stage is the leaving stage which occurs when $s> N-m_{\mathrm{p}}$ and shows the free energy change $\Delta F(s) = (s-N+m_{\mathrm{p}})\Delta \mu _{\mathrm{ps}}$. Here the chemical potential difference between the pore and the semi-space, $\Delta \mu _{\mathrm{ps}}$, is negative. The chain is confronted to a thermodynamic driving $|\Delta \mu _{\mathrm{ps}}|/\sigma$ and the leaving time is $\tau _{\mathrm{leav}}\simeq m_{\mathrm{p}}\left( \frac{k_{{\mathrm{B}}}T}{|\Delta \mu _{\mathrm{ps}}|}\right) \Delta {\mathfrak {t}}_0$, which is generally short and negligible.

**Figure 1:**
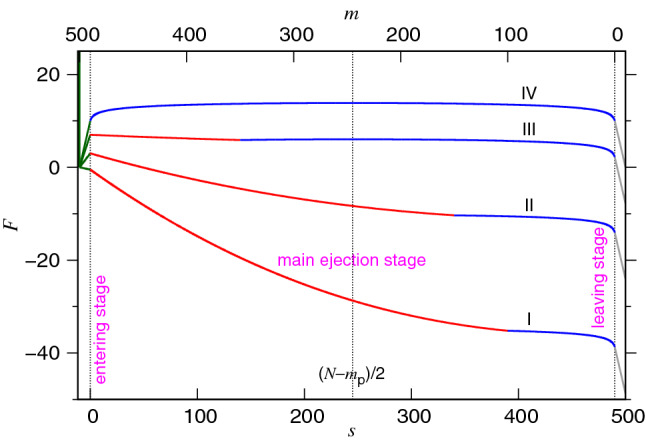
Free energy *F* as a function of the state variables *s* (the bottom axis) and *m* (the top axis). We choose $N=500$ and $m_{\mathrm{p}}=10$ to draw the sketch. In the entering and the leaving stages, the free energy curves are colored in dark-green and gray, respectively. In the main ejection stage, the curve is split into two sections: one in red color representing the confined stage and the other in blue color representing the non-confined stage. There exist four kinds of variations, denoted by I, II, III, and IV near the curves.

According to the physical pictures described above, the free energy landscape of an ejection process in the space of the state variables *s* and *m* is sketched in Fig. 1. It can be classified into four situations, indicated by the roman numerals I to IV in the figure. Curve I presents the free energy change in an ejection process beginning with an initial volume fraction $\phi _0$ higher than the local volume fraction $\phi _{\mathrm{p}}$ of a monomer when it is presented in the pore. In this situation, the free energy decreases monotonically, from the entering stage, through the main ejection stage, to the leaving stage. The main ejection stage is subdivided into the confined and the non-confined stages, shown in red and blue colors, respectively. Curve II, similar to Curve I, depicts a large $\phi _0$ situation but the value of $\phi _0$ is smaller than $\phi _{\mathrm{p}}$. As a consequence, the free energy increases in the entering stage, which is a Kramers escape problem. The free energy then decreases monotonically in the following stages. The point demarcating the confined and the non-confined stages locates at $s=s_* \equiv N-m_{\mathrm{p}}-N_*$ which is right to the middle $(N-m_{\mathrm{p}})/2$. To meet the situation, the chain length must fulfill the condition $(N-m_{\mathrm{p}})> 2N_*$. If the demarcating happens before reaching the middle of the process, the free energy does not monotonically decrease. There is a uphill to be mounted between $s=s_*$ and $s=(N-m_{\mathrm{p}})/2$. This is the situation III occurring when $N_*<(N-m_{\mathrm{p}})<2N_*$, where a second escape is required in the non-confined stage. If $N-m_{\mathrm{p}}$ is smaller than $N_*$, the free energy looks similar to Curve IV. In this situation, the two escapes joint together, since the beginning at $s=-m_{\mathrm{p}}$ to the middle of the process at $s=(N-m_{\mathrm{p}})/2$. Once escaping the energy summit, the chain arrives the downhill side of the free energy for the rest of the process.

Based upon the above analysis, the non-confined stage can be distinguished into the thermal escape and the entropic pulling substages. The required time $\tau _{\mathrm{ej}}$ for the main ejection stage is thus a sum of the three components: $\tau _1$ the time spent for the confined stage, $\tau _{\mathrm{2E}}$ the time needed for the thermal escape substage, and $\tau _{\mathrm{2P}}$ the one for the entropic pulling substage. The predicted time scales for $\tau _1$, $\tau _{\mathrm{2E}}$, and $\tau _{\mathrm{2P}}$ are given in Table 1.

**Table 1:** Predicted time scales $\tau _1$, $\tau _{\mathrm{2E}}$, and $\tau _{\mathrm{2P}}$ in the main ejection stage.

Chain length	Confined stage	Non-confined stage	
	$\tau _1/\Delta {\mathfrak {t}}_0$	$\tau _{\mathrm{2E}}/\Delta {\mathfrak {t}}_0$	$\tau _{\mathrm{2P}}/\Delta {\mathfrak {t}}_0$
$N-m_{\mathrm{p}}< N_*$	0	$N^{2+x_1}I_{1}(0)+N^{2+y_{\mathrm{2E}}}I_{\mathrm{2E}}(0)$	$\frac{1}{1+z_{\mathrm{2P}}} \left( \frac{N}{2}\right) ^{1+z_{\mathrm{2P}}}$
$N_*\le N-m_{\mathrm{p}}\le 2N_*$	$\frac{N^{x_1}}{z_1-1}N_*^2\left[ 1-\left( \frac{N_*}{N}\right) ^{z_1-1}\right]$	$N^{2+x_1}I_{1}({\tilde{s}}_*)+N^{2+y_{\mathrm{2E}}}I_{\mathrm{2E}}({\tilde{s}}_*)$	$\frac{1}{1+z_{\mathrm{2P}}} \left( \frac{N}{2}\right) ^{1+z_{\mathrm{2P}}}$
$N-m_{\mathrm{p}}> 2N_*$	$\frac{N^{x_1}}{z_1-1}N_*^2\left[ 1-\left( \frac{N_*}{N}\right) ^{z_1-1}\right]$	0	$\frac{1}{1+z_{\mathrm{2P}}} N_*^{1+z_{\mathrm{2P}}}$

Three situations are distinguished for the chain length: $N-m_{\mathrm{p}}<N_*$, $N_*\le N-m_{\mathrm{p}}\le 2N_*$, and $N-m_{\mathrm{p}}> 2N_*$ where $N_*\sim (D/\sigma )^{1/\nu }$ is the critical chain length. The ejection time $\tau _{\mathrm{ej}}$ is a sum of the three time scales.

## Simulation model and settings

**Figure 2:**
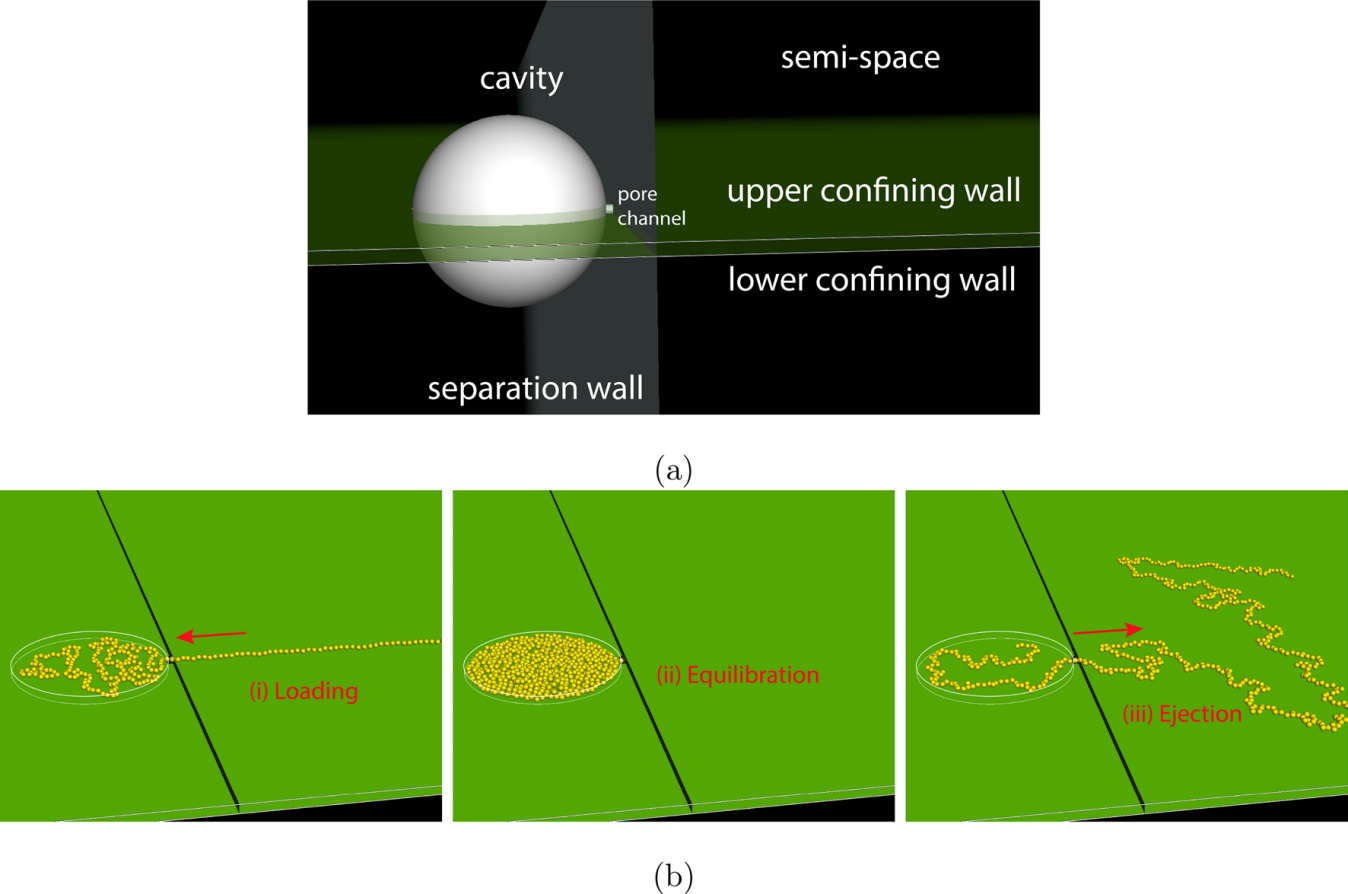
(**a**) Setting of the quasi two-dimensional ejection system, which is realized by adding an upper and a lower confining wall on top of the three-dimensional “spherical cavity–pore channel–semispace” ejection system. (**b**) Snapshots of the system at the three processing phases: (i) loading, (ii) equilibration, and (iii) ejection. The chain has 512 beads and the volume fraction of beads after fully loaded into the cavity is 0.3.

In this work, we investigate the ejection process of a polymer in a quasi two-dimensional space and verify the scaling theory. We confine the polymer in a disklike cavity and study its ejection, through a small pore situated on the side of the cavity, into an outer slit space. The disklike cavity and the slit space are prepared by adding two parallel confining walls on top of the “spherical cavity–pore channel–semispace” system, as shown in Fig. 2a, which restricts the motion of polymer in a quasi two-dimensional space. The polymer is modeled by a bead-spring chain and loaded into the cavity by a pumping process. The loaded chain is then equilibrated with the head monomer being fixed at the pore entrance, which blocks the exit of the chain. The ejection is started by freeing the head monomer and the chain ejects spontaneously out of the cavity through the pore. The snapshots of the system are given in Fig. 2b for the three processing phases: (i) loading, (ii) equilibration, and (iii) ejection.

The chain beads interact with each others via a Lennard-Jones (LJ) 12-6 potential $U_{\mathrm{ex}}(r)= 4\varepsilon \left[ \left( \frac{\sigma }{r}\right) ^{12} - \left( \frac{\sigma }{r} \right) ^{6} \right]$, shifted and truncated at the minimum point $r_{\mathrm{c}}=\root 6 \of {2} \sigma$, where *r* is the distance between a pair of beads, and $\varepsilon$ and $\sigma$ are the interaction strength and length, respectively. The beads are connected by springs and form linear architecture. The potential of spring is $U_{\mathrm{sp}}(b)=\frac{1}{2} k (b-b_0)^2$ where *k* is the spring constant and $b-b_0$ is the stretching length. The beads have repulsive interaction with the the confining wall via a LJ 9-3 potential, $U_{\mathrm{w}}(r)= \varepsilon _{\mathrm{w}} \left[ \frac{2}{15} \left( \frac{\sigma _{\mathrm{w}}}{r}\right) ^{9} - \left( \frac{\sigma _{\mathrm{w}}}{r} \right) ^{3} \right]$, shifted and truncated at $r=\root 6 \of {\frac{2}{5}} \sigma _{\mathrm{w}}$. We set $\varepsilon _{\mathrm{w}}=3.0\varepsilon$, $\sigma _{\mathrm{w}}=\sigma$, $k=600\varepsilon /\sigma ^2$, and $b_0=\sigma$. The temperature *T* is controlled by using Langevin thermostat^54^ and set to be $1.0\,\varepsilon /k_{\mathrm{B}}$. The relaxation time for the temperature control is $1.0\,\sigma \sqrt{{\mathfrak {m}}/\varepsilon }$. Here $k_{\mathrm{B}}$ is the Boltzmann constant and ${\mathfrak {m}}$ is the mass of a bead. Under this setting, the wall potential attains the value of thermal energy $k_{\mathrm{B}}T$ at $r=0.76\sigma$. It implies that the wall has an effective thickness equal to $0.26\sigma$ because the bead radius is $0.5\sigma$. Therefore, we set the cavity wall on a sphere of diameter $D_{\mathrm{C}}=D+0.52\sigma$, which produces the desired disk diameter *D* in the study. The two parallel walls are placed in a way to create a confining space with the gap height equal to $H=1.5\sigma$. The pore channel is open on the side of the disklike cavity and modeled by a cylinder with the effective diameter $d_{\mathrm{p}}=1.5\sigma$ and length $\ell _{\mathrm{p}}=1.0\sigma$.

We vary the number of beads on a chain from $N=8$ to 1024, as a power of 2, and denote it by $N_{g_N}=2^{g_N}$. The diameter *D* is varied to produce the desired initial volume fraction $\phi _0$ of monomers in the cavity at a given *N*. It can be shown that $\phi _0$ is related to *N*, *D* and *H* by

6 $$\begin{aligned} \phi _0=N\left( \frac{\sigma }{D}\right) ^2 \times \left[ \frac{2\sigma }{3H\left( 1-\frac{1}{3}\left( \frac{H}{D}\right) ^2\right) }\right] \end{aligned}$$

We see that $\phi _0$ is about equal to the two-dimensional volume fraction $N\left( \frac{\sigma }{D}\right) ^2$ multiplying the factor $\frac{2\sigma }{3H}$ if $H\ll D$. We study the cases of the initial volume fraction equal to $\phi _{0,g_F}=0.3 \times 2^{-g_F}$ where the generation number $g_F$ is varied from 0 to 10. The corresponding diameter *D* can be thus calculated by the formula

7 $$\begin{aligned} D_{g_D}=\sigma \sqrt{\frac{2^{g_D}}{0.3}}\times \sqrt{\frac{2\sigma }{3H}+\frac{1}{3}\left( \frac{H}{\sigma }\right) ^2\cdot 0.3\times 2^{-g_D}}. \end{aligned}$$

Here $g_D=g_N+g_F$ is the generation number for *D*. Using the generation numbers allows us to investigate the scaling behaviors in a logical way. For each studied ($N_{g_N}$, $D_{g_D}$, $\phi _{0,g_F}$) case, five hundred independent runs are performed. The simulation trajectories are recorded for post analysis. The case with $g_F=\infty$ is also studied for comparison. It simulates the situation that a chain is transported across a line barrier through a small pore in a quasi two-dimensional space. To prevent falling of the entire chain back into the cavity, especially when *D* is large, we have set a reflective wall at the pore entrance which interacts only with the head monomer to bounce it back into the pore. Therefore, searching for the pore entrance from the inside of the cavity for the head monomer is not involved in this study. We remark that there is no external stimulus applied in the model. The ejection of chain is effectuated spontaneously because of the favor of thermodynamics. However, chemical potential difference does exist in the different regions of the system. The pore channel has a small diameter and thus the chemical potential for a monomer presenting inside it is generally higher than the one presenting in the cavity and in the outer semi-space, determined basically by the local volume fraction. In a real case, some mechanism or extra chemical potential gradient may exist inside the pore to help progresses of an ejection. We do not study the effect here.

In this paper, we choose ${\mathfrak {m}}$, $\sigma$, $\varepsilon$ to be the mass, length, energy units, respectively. A physical quantity will be reported only by its value in the following text, without explicitly mentioning the unit. For example, the ejection time “$\tau _{\mathrm{ej}}=100.0$” means $\tau _{\mathrm{ej}}=100.0\, t_{\mathrm{u}}$ where $t_{\mathrm{u}}=\sigma \sqrt{{\mathfrak {m}}/\varepsilon }$ is the time unit. The velocity “$V_{\mathrm{ej}}=3.0$” means $V_{\mathrm{ej}}=3.0\, \sigma /t_{\mathrm{u}}$.

## Results

### Ejection speed

The ejection kinetics is directly studied by calculating the averaged ejection speed $\langle V_{\mathrm{ej}} \rangle$. The speed is not constant and varies a lot during a process. As we will see, the largest ejection speed in a process could be hundred times, or even more, larger than the smallest speed, depending on the condition of ejection. Knowing the variation of $\langle V_{\mathrm{ej}} \rangle$ allows to understand how a chain is ejected at any moment of a process.

**Figure 3:**
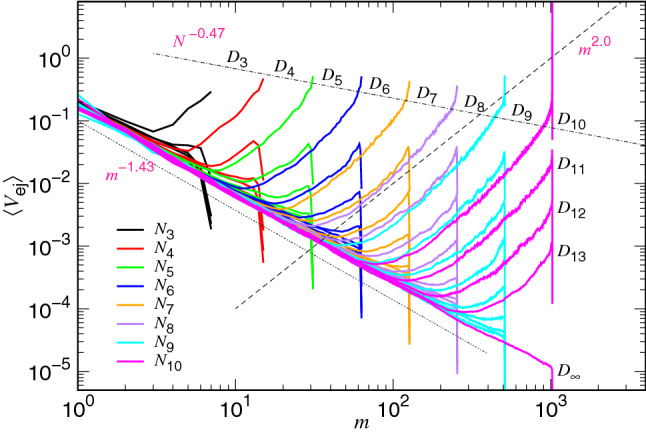
Averaged ejection speed $\langle V_{\mathrm{ej}}\rangle$ as a function of the number of monomers *m* in the cavity. The chain length *N* is given in the legend by the formula $N_i=2^i$. The diameter *D* of cavity is indicated on the top-right corner of the family curves which share the same $D_g$ value, calculated by using Eq. (7). The dashed line $m^{2.0}$ and the dotted line $m^{-1.43}$ show the scaling behavior of the ejection speed in the confined and the non-confined stages, respectively. The dash-dotted line $N^{-0.47}$ indicates the decreasing trend of the initial speed with the chain length.

The speed $\langle V_{\mathrm{ej}} \rangle$ at a state *m* is equal to $\sigma /W(m)$ where $\sigma$ is the length of a segment and *W*(*m*) is the mean dwelling time at the state. We recall that *m* is the number of the monomers remaining in the cavity. The variation of *W* as a function of *m* is also called the waiting time function, which can be calculated from the simulations by measuring the duration time of a chain staying at each state *m*. Figure 3 presents $\langle V_{\mathrm{ej}} \rangle$ as a function of *m* in an ejection process for different chain lengths and cavity diameters. Because *m* decreases with time, the $\langle V_{\mathrm{ej}} \rangle$ curve should be looked from the right to the left in the figure to follow the direction of time arrow. The chain length *N* is varied from $N_5=32$ to $N_{10}=1024$ and the cavity diameter *D* is varied from $D_3$ to $D_{18}$ in this study, where the values of $D_{g}$ are given by Eq. (7). The cases with infinite diameter $D=D_{\infty }$ are also studied for references, which corresponds to the translocation of polymer in a two-dimensional space.

We observe two main variational behaviors in a typical ejection process: $\langle V_{\mathrm{ej}} \rangle$ decreases at the confined stage and turns to show increasing behavior at the non-confined stage. On the log-log plot, the two variations follows essentially the scaling $m^{z_1}$ and $m^{-z_{\mathrm{2P}}}$, respectively, where the predicted exponents are $z_1=(d\nu -1)^{-1}=2.0$ and $z_{\mathrm{2P}}=2\nu =1.5$ by setting $d=2$ and $\nu =0.75$. The two values are significantly larger than the exponents for the three-dimensional ejection, $z_1=1.25$ and $z_{\mathrm{2P}}=1.2$^39,40^.

**Figure 4:**
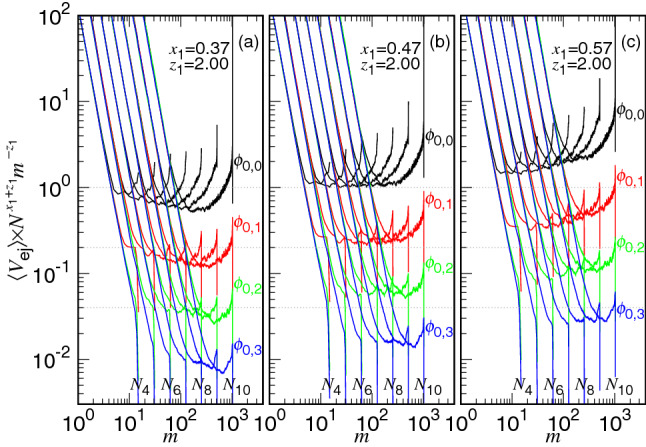
Rescaled speed $\langle V_{\mathrm{ej}} \rangle \times N^{x_1+z_1} m^{-z_1}$ vs. *m*, by setting (**a**) $x_1=0.37$, (**b**) $x_1=0.47$, and (**c**) $x1=0.57$. The exponent $z_1$ is assumed 2.0. The chain length $N_i=2^i$ is indicated near the bottom of the figure close to the curve. The initial volume fraction is indicated on the right side of the set of the curves sharing the same $\phi _0$ value, calculated by the formula $\phi _{0,j}=0.3\times 2^{-j}$.

The kinetics equation in Eq. (2) implies that $\langle V_{\mathrm{ej}} \rangle$ should be identical to each other for different *N* values at a given state *m* if *D* (and hence $N_*$) is fixed. However, the simulations reveal that the speed curve for a longer chain is evidently lower than the one for a shorter chain. To rectify it, we have introduced a chain length dependence for the friction coefficient, $\eta \sim \eta _0 N^{x_1}$, to reduce the ejection speed. The reason for the increase of $\eta$ with *N* will be explained later, after Fig. 7. An exponent of $x_1=1/3$ has been found in the study of the three-dimensional ejection^39,40^. Here the scaling argument is extended and $x_1$ is anticipated to be 1/2 in our two-dimensional case. We can see that the ejection speed at starting (near $m=N$) descends with increasing the chain length as $\phi _0$ is fixed. It follows *grosso modo* a scaling $N^{-x_1}$, indicated by a dash-dotted line in the figure. In order to determine the value of $x_1$, we plot the rescaled speed $\langle V_{\mathrm{ej}} \rangle \times N^{x_1+z_1} m^{-z_1}$ in Fig. 4, by setting different values for $x_1$. Under the fixed-$\phi _0$ condition, the rescaled speed at the confined stage is expected to be constant for different chain lengths because

8 $$\begin{aligned} V_{\mathrm{ej}}\sim \frac{m^{z_1}}{N^{x_1} N_*^{d \nu z_1}} v_0 \sim \frac{m^{z_1}}{N^{x_1} (D/\sigma )^{d z_1}} v_0 \sim \frac{m^{z_1}}{N^{x_1} (N/\phi _0)^{z_1}} v_0 \end{aligned}$$

which is derived from Eq. (2) together with the scaling assumption for $\eta$. Here $v_0=\sigma /\Delta {\mathfrak {t}}_0$ is the characteristic speed, served as the unit.

As we can see, the rescaled curve is leveled off for a while as $m>N_*$ and becomes bent-up as *m* approaches *N*. The leveling-off value is found to be independent of *N* by setting $x_1=0.47$ at the four studied $\phi _0$ values (refer to Panel (b)). Decreasing or increasing $x_1$ tilts the leveling to the right or the left, as shown in the panels (a) and (c) of the figure. It gives the best estimate for the $x_1$ exponent. We will come back to explain later why the curve is curved up near $m=N$.

**Figure 5:**
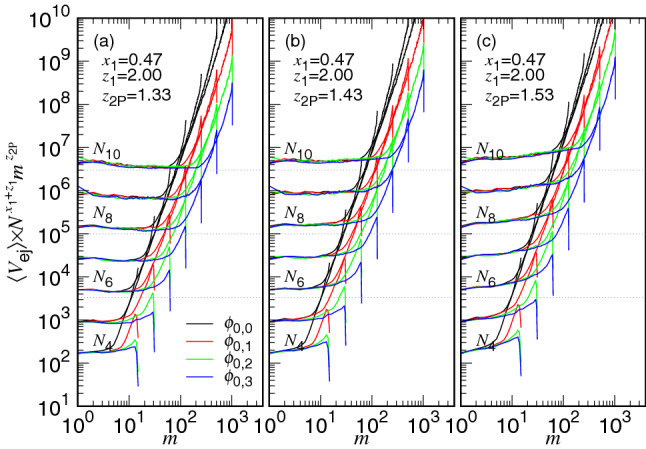
$\langle V_{\mathrm{ej}} \rangle \times N^{x_1+z_1} m^{z_{\mathrm{2P}}}$ versus *m*, by setting (**a**) $z_{\mathrm{2P}}=1.33$, (**b**) $z_{\mathrm{2P}}=1.43$, and (**c**) $z_{\mathrm{2P}}=1.53$, with $x_1=0.47$ and $z_1=2.0$. The chain length $N_i=2^i$ is indicated on the left near the curve. The black, red, green, and blue curves have $\phi _0=\phi _{0,j}$ with $j=0$, 1, 2, 3, respectively.

If we plot $\langle V_{\mathrm{ej}} \rangle \times N^{x_1+z_1} m^{z_{\mathrm{2P}}}$ versus *m*, the rescaled speed will be leveled off in the non-confined stage ($m<N_*$), according to Eq. (5). The rescaling has still involved the factor $N^{x_1+z_1}$ in the multiplication, which guarantees the rescaled curves coinciding in the confined stage at a given $\phi _0$ value. Figure 5 shows the search for the value of the exponent $z_{\mathrm{2P}}$. We find that setting $z_{\mathrm{2P}}=1.43$ (see Panel (b)) produces the best overall horizontal lines in the non-confined stage. Decreasing or increasing $z_{\mathrm{2P}}$ inclines the horizontal lines to the right (in Panel (a)) or to the left (in Panel (c)).

**Figure 6:**
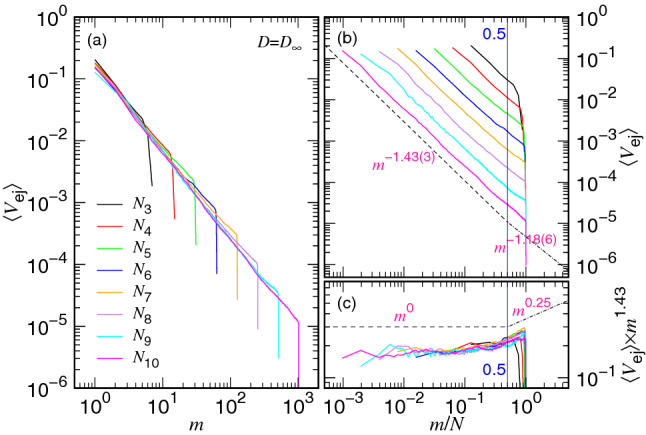
(**a**) $\langle V_{\mathrm{ej}} \rangle$ vs. *m*, (**b**) $\langle V_{\mathrm{ej}} \rangle$ vs. *m*/*N*, and (**c**) $\langle V_{\mathrm{ej}} \rangle \times m^{1.43}$ vs. *m*/*N*, at $D=D_{\infty }$ for various chain lengths. The $N_i$ value is given by the formula $2^i$ and the color legend can be read in Panel (**a**).

To understand the details of the speed scaling in the non-confined stage, we plot $\langle V_{\mathrm{ej}} \rangle$ as a function of *m* for $D=\infty$ in Fig. 6a. We can see that the ejection increases its speed with decreasing *m* and the speed curves follow a universal path when *m* becomes small. According to the physical pictures described in "General ejection theory in a *d*-dimensional space" section, the process stays entirely in a non-confined situation. In the first half part of the process ($m>0.5N$), the free energy of the system goes uphill and the “ejection” is a Kramers escape problem. We have derived a formula for the escape time (refer to the case $N-m_{\mathrm{p}}<N_*$ in Table 1) and the ejection speed in this part exhibits an overall scaling with the exponent equal to $-z_{\mathrm{2E}}$. In the second half part of the process, the number *m* of the monomers in the cavity becomes smaller than the one *s* in the semi-space. At this moment, the ejection is driven by the entropic pulling of the chain segments from the outside. The free energy goes downhill, and the ejection speed is described by Eq. (5) and scales as $V_{\mathrm{ej}}\sim m^{-z_{\mathrm{2P}}}$. Figure 6b shows the variation of $\langle V_{\mathrm{ej}} \rangle$ vs. *m*/*N*. By performing non-linear regression fits in the two regions, $0.01<m/N<0.5$ and $0.5<m/N<0.95$, separately, for the chain lengths from $N=64$ to 1024, we report the best estimates for the two exponents: $z_{\mathrm{2P}}=1.43(3)$ and $z_{\mathrm{2E}}=1.18(6)$. To see the difference of the exponents, we have plotted the rescaled speed $\langle V_{\mathrm{ej}}\rangle \times m^{1.43}$ versus *m*/*N* in Fig. 6c. The rescaled curves are horizontal in the region $m/N<0.5$ and turn to show $m^{0.25}$ scaling when $m/N>0.5$.

Despite the fact that the ejection speed can be well described by the kinetics equation Eq. (5) in the non-confined stage, a large bending-up effect, away from the predicted scaling $V_{\mathrm{ej}}\sim m^{z_{1}}$ for the confined stage, has been observed in Fig. 3 particularly when the *m* value is close to *N*. The deviation occurs when the monomers are very dense in the cavity and the effect can be taken into account by considering the “second” virial term for the free energy^55^

9 $$\begin{aligned} F\sim k_{{\mathrm{B}}}T\left[ \left( \frac{m}{N_*}\right) ^{z_1+1} +\frac{B_2}{2} \left( \frac{m}{N_*}\right) ^{2(z_1+1)} \right] \end{aligned}$$

where $B_2$ is the 2nd virial coefficient for the semidilute polymer solution. The kinetics equation for *m* at the confined stage is thus rectified:

10 $$\begin{aligned} \frac{\mathrm{d}m}{\mathrm{d}t}\sim -\frac{z_1+1}{\Delta {\mathfrak {t}}_0 N^{x_1}} \left( \frac{m^{z_1}}{N_*^{z_1+1}}\right) \left[ 1+ B_2 \left( \frac{m}{N_*}\right) ^{z_1+1}\right] . \end{aligned}$$

The second virial term boosts the growth of the speed which bends the curve up in the log-log plot.

To verify the theory, we combine directly Eqs. (10) and (5) and approximate the ejection speed of the process by using a single equation

11 $$\begin{aligned} V_{\mathrm{ej}}\simeq v_0 \left\{ A_1\left( \frac{m^{z_1}}{N^{x_1}(D/\sigma )^{dz_1}}\right) \left[ 1+ B_2\left( \frac{m^{z_1+1}}{(D/\sigma )^{dz_1}}\right) \right] +A_2 m^{-z_{\mathrm{2P}}} \right\} \end{aligned}$$

**Figure 7:**
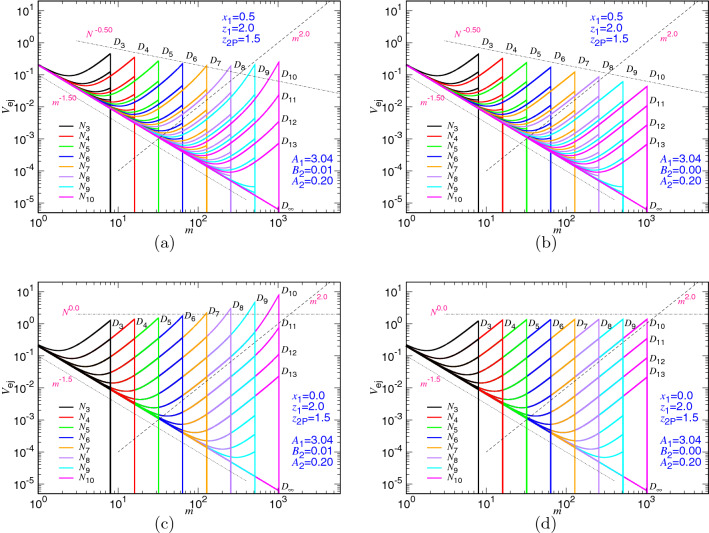
Ejection speed $V_{\mathrm{ej}}$ predicted by Eq. (11) for various *N* and *D* with the parameters: (**a**) $A_1=3.04$, $B_2=0.01$, $A_2=0.20$, and (**b**) $A_1=3.04$, $B_2=0$, $A_2=0.20$. The exponents $x_1$, $z_1$, and $z_{\mathrm{2P}}$ are set to the two-dimensional values, 0.5, 2.0, and 1.5, respectively. The chain length $N_i$ is $2^i$ and the cavity diameter $D_g$ is calculated by Eq. (7). Panels (**c**) and (**d**) present the same plots as Panels (**a**) and (**b**), respectively, except that $x_1$ is set to zero, which turns off the chain length dependence $N^{x_1}$ in Eq. (11).

Here $A_1$ and $A_2$ are two prefactors which give the required weightings for the two scaling speeds derived separately in the confined and non-confined stages. We have utilized the scaling relation $D \sim \sigma N_*^{\nu }$ to substitute $N_*$ in the equation. Having the approximated speed equation on hand, we are able to perform non-linear regression fits for the simulation data and find $A_1=3.04$, $B_2=0.010$, and $A_2=0.20$. We make the plot of Eq. (11) in Fig. 7a by plugging in the three fitting parameters for the set of the studied *N* and *D*, using the theoretical exponents $x_1=0.5$, $z_1=2.0$, and $z_{\mathrm{2P}}=1.5$.

Astonishing agreements in many features of the speed curves are observed between the theoretical plot and the simulations (Fig. 3). For example, the ejection speed in the non-confined stage is well described by the scaling $m^{-z_{\mathrm{2P}}}$, while in the confined stage, the curve curves up from the scaling line $m^{z_1}$ as *m* approaches *N*. The initial speed for different chain lengths does not follow well the predicted decreasing $N^{-x_1}$, as shown in Fig. 3. It is unlike the three-dimensional case^40^ where the initial speed follows essentially $N^{-0.33}$. To see more clearly the impact of the second virial term on the two-dimensional ejection, we replot Eq. (11) by setting $B_2=0$ in Fig. 7b, which switches off the second virial. We see right away that the two defectives are gone. The ejection speed in the confined stage and the initial speed both follow the desired scalings. It demonstrates the strong influence of the second virial term for the two-dimensional case, compared to the three-dimensional one.

Figure 7c,d present the change of the speed profiles with and without involving the second virial, respectively, under the condition that there is no chain length dependence $N^{x_1}$ in the denominator of the confined stage in Eq. (11). We can see that the $V_{\mathrm{ej}}$ curves are collapsed for different *N* at the same *m*-state value as *D* is fixed, and the initial speed stops decreasing with *N*. The profiles look very different to the ones observed in Fig. 3. It shows the necessity to have the $N^{-x_1}$ dependence in the confined stage to describe correctly the ejection speed.

The appearance of the dependence reflects the fact that the effective friction coefficient should scale like $\eta \sim \eta _0 N^{x_1}$. The results suggest that the energy dissipation in the confined stage does not come only from the traversing monomer in the pore but also from certain following monomers, owing to the chain connectivity. These monomers move with a similar speed $V_{\mathrm{ej}}$ in the cavity and are estimated to have a length of about the cavity size *D*, or equivalently about $m^{1/d}$ monomers. The monomers just ejected to the outer space participate the energy dissipation too. We estimate about $(N-m)^{1/d}$ such monomers to give up their speed $V_{\mathrm{ej}}$ at that moment. Because the ejection speed is derived from the balancing equation $\mathrm{d}F/\mathrm{d}t=-\eta V_{\mathrm{ej}}^2$ where the dissipation is presumed to occur only at the pore, the effective friction coefficient $\eta$ is thus $\eta _0(m^{1/d}+(N-m)^{1/d})$, which scales roughly like $\eta _0 N^{1/d}$ if *N* is large. As a consequence, the ejection speed exhibits the chain length dependence with $x_1=1/d$ in the confined stage.

Concerning the effective friction coefficient in the non-confined stage, we expect that $\eta$ scales as $\eta _0 m^{y_{\mathrm{2P}}}$. The key difference with in the confined stage is that all the monomers on the cis side now participate the energy dissipation. These monomers are not confined by the cavity and hence show a net movement toward the pore with the drift speed estimated to be $v_d\sim \frac{\mathrm{d}R_m}{\mathrm{d}t} \sim \frac{\mathrm{d}(\sigma m^{\nu })}{\mathrm{d}t} \sim m^{\nu -1} \sigma \frac{\mathrm{d}m}{\mathrm{d}t} \sim m^{\nu -1} V_{\mathrm{ej}}$. Under this situation, the rate of the energy dissipation is dominated by the *m* monomers on the cis side and about $m \cdot \eta _0 v_d^2\sim \eta _0 m^{2\nu -1}V_{\mathrm{ej}}^2$. Therefore, the exponent $y_{\mathrm{2P}}$ is $2\nu -1$, from which we obtained the ejection speed $V_{\mathrm{ej}}\sim v_0 m^{-z_{\mathrm{2P}}}$ with $z_{\mathrm{2P}}=1+y_{\mathrm{2P}}=2\nu$.

**Figure 8:**
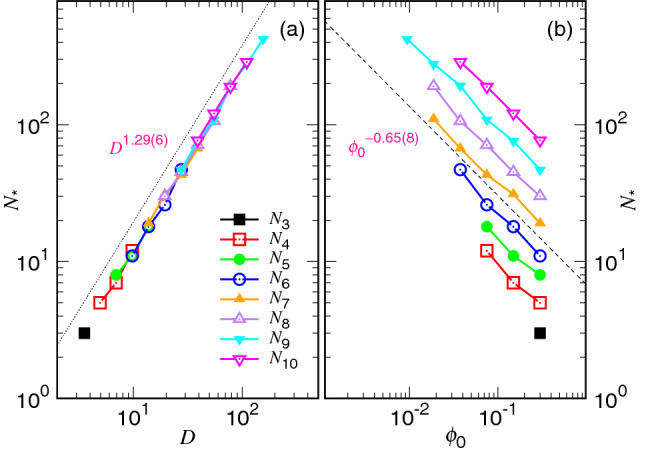
(**a**) Critical monomer number $N^*$ as a function of the cavity diameter *D* for various chain lengths *N*. (**b**) $N_*$ as a function of $\phi _0$ for different *N*. The color legend for *N* is given in Panel (**a**) where $N_i=2^i$.

Before ending this subsection, we study the scaling behavior of the critical number $N_*$. The number is determined by searching for the position of the minimum ejection speed in Fig. 3. The results are given in Fig. 8.

We find that $N_*$ scales as $D^{1.29(6)}$ in Panel (a), and is independent of *N* because the data obtained from different chain lengths lie on a universal line. It agrees with our assumption that $D\sim \sigma N_*^{\nu }$, which implies $N_*\sim (D/\sigma )^{1/\nu }$. Since $\nu$ is about 0.75 for a two-dimensional space, an exponent value around 4/3 is expected on the figure. Concerning the scaling against the initial volume fraction, because $\phi _0 \sim N (\sigma /D)^d$, we anticipate $N_*\sim (N/\phi _0)^{1/(d\nu )}$. It gives $N_*\sim (N/\phi _0)^{2/3}$. This is exactly what we have observed in the panel (b). The sets of data decrease in a parallel manner for different chain lengths. The longer chain possesses a larger $N_*$ value at a given $\phi _0$.

### Decrease of number of monomers in the cavity

**Figure 9:**
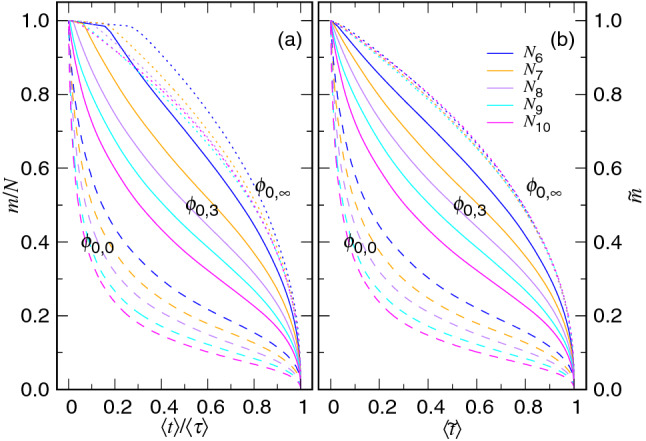
(**a**) *m*/*N* vs. $\langle t\rangle /\langle \tau \rangle$ and (**b**) ${\tilde{m}}$ vs. $\langle {\tilde{t}}\rangle$ at the three initial volume fractions: $\phi _{0,0}\equiv 0.3$ (in dashed line), $\phi _{0,3}\equiv 0.3\times 2^{-3}$ (in solid line), and $\phi _{0,\infty }\equiv 0.0$ (in dotted line). The chain length can be read in the legend of Panel (**b**) where $N_i\equiv 2^{i}$.

We study the variation of the number of monomers *m* in the cavity during an ejection process. Because the mean time $\langle t \rangle$ to reach a state *m* depends on the chain length and the initial volume fraction, we rescale the time and the number of the monomers by the total time $\langle \tau \rangle$ and *N*, respectively, in order to compare different simulations. Figure 9a presents the results of *m*/*N* vs. $\langle t\rangle /\langle \tau \rangle$ at three selected $\phi _0$ values for different chain lengths.

We observe that the number of the monomers does not decrease smoothly. A plateau region appears at the beginning of the curve, as $\phi _0$ is not large, with the value of *m* descended by 1. The descending number corresponds to the pore length $\ell _{\mathrm{p}}=1.0$. The phenomena have been identified in the three dimensional ejection and can be analogous to a nucleation process^39,40^. Once the first monomer escapes the pore channel and reaches the semi-space, the ejection can take place in a smooth way without stalling. Therefore, the required descending number 1 can be regarded as the “critical nucleus size” here for the further growth of the number of the monomers in the semi-space. At large $\phi _0$ value such as 0.3, the plateau region does not appear and the curve decreases directly from the beginning.

To understand properly the decreasing behavior, we trim the critical nucleus size $m_{\mathrm{n}}$ off and replot the curves using the two trimmed variables: $\langle {\tilde{t}}\rangle =(\langle t\rangle -\tau _{\mathrm{n}})/(\langle \tau \rangle -\tau _{\mathrm{n}})$ and ${\tilde{m}} = (m - m_{\mathrm{n}})/(N-m_{\mathrm{n}})$ where $m_{\mathrm{n}}$ is one in this case and $\tau _{\mathrm{n}}$ is the the nucleation time which is equal to the time $\langle \tau _{\mathrm{ent}}\rangle$ spent in the entering stage for the heading monomer to go across the pore. We can see in Fig. 9b that the curves now decrease smoothly without stalling. At $\phi _0=\phi _{0,\infty }$, the trimmed curves are overlapped with each other, following a master descending curve for different chain lengths. With $\phi _0$ being increased, the concave curves turn to show covexness in the small $\langle \tilde{t\rangle }$ region. The longer the chain length, the faster the descending will be.

**Figure 10:**
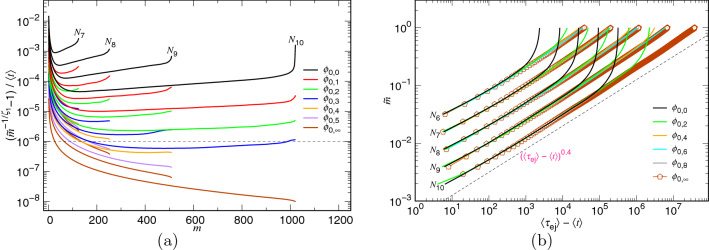
(**a**) $({\tilde{m}}^{-1/\zeta _1}-1)/\langle t\rangle$ vs. *m* by setting $\zeta _1=1.0$ and (**b**) ${\tilde{m}}$ vs. $\langle \tau _{\mathrm{ej}}\rangle - \langle t\rangle$. The $\phi _0$ value can be read from the legends while the chain length *N* is indicated directly near the corresponding curves.

The variation of *m* with time can be predicted by solving the kinetics equation for the confined stage, Eq. (2), with the initial condition $m=N$ at $t=0$, and reads as

12 $$\begin{aligned} {\tilde{m}} \simeq \left( 1+\frac{t}{t_0} \right) ^{-\zeta _1} \end{aligned}$$

where $t_0 \sim \zeta _1 N_*^{1+z_1}N^{1-z_1+x_1}\Delta {\mathfrak {t}}_0$ and $\zeta _1=(z_1-1)^{-1}$. For the non-confined stage, the variation can be solved from Eq. (5), with the “finial” state condition $m=0$ at $t=\tau _{\mathrm{ej}}$. We obtain

13 $$\begin{aligned} {\tilde{m}} \simeq \frac{1}{N}\left( \frac{1}{\zeta _2\Delta {\mathfrak {t}}_0}\right) ^{\zeta _2} \left( \tau _{\mathrm{ej}}- t \right) ^{\zeta _2} \end{aligned}$$

where $\zeta _2=(z_2+1)^{-1}$. To understand how good the prediction is, we plot the two functions: $({\tilde{m}}^{-1/\zeta _1}-1)/\langle t\rangle$ vs. *m* and ${\tilde{m}}$ vs. $\langle \tau _{\mathrm{ej}}\rangle - \langle t\rangle$, from the simulations in Fig. 10. The previous function is expected to be $1/t_0$, which is constant for *m* close to *N* at large $\phi _0$. The latter should exhibit a power-law growth with an exponent $\zeta _2$ in the small $\langle \tau _{\mathrm{ej}}\rangle -\langle t\rangle$ region.

Because the predicted $z_1$ exponent is 2.0, we set $\zeta _1=1.0$ in the Panel (a) of the figure. We can see that the $({\tilde{m}}^{-1/\zeta _1}-1)/\langle t\rangle$ curves are essentially horizontal over a broad range of *m* in the confined stage ($m>N_*$) as $\phi _0$ is large for the chain length varied from $N_7$ to $N_{10}$. Figure 10b shows that ${\tilde{m}}$ follows a scaling relation $(\langle \tau _{\mathrm{ej}}\rangle -\langle t\rangle )^{0.4}$. The exponent $\zeta _2=0.4$ gives $z_2=1.5$, which corresponds well to the predicted value and the findings in Fig. 3. The exponents $\zeta _1$ and $\zeta _2$ found here are significantly smaller than the ones for the three-dimensional ejection: $\zeta _1=4.0$ and $\zeta _2=0.454$^40^.

**Figure 11:**
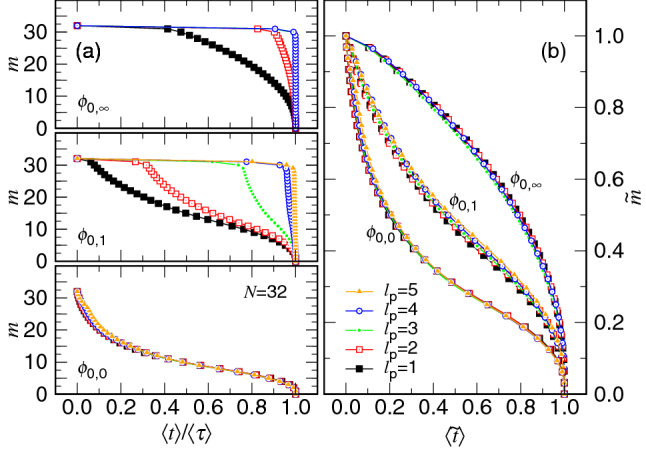
(**a**) *m* vs. $\langle t\rangle /\langle \tau \rangle$ for various pore length $\ell _{\mathrm{p}}$ at $\phi _0=\phi _{0,\infty }$, $\phi _{0,1}$, and $\phi _{0,0}$. (**b**) Time variation of the number of monomers plotted in the normalized coordinates ${\tilde{m}}$ and $\langle {\tilde{t}}\rangle$ at the three $\phi _0$. The value of $\ell _{\mathrm{p}}$ can be read in the legend.

To understand the stalling for the decrease of $\langle m\rangle$ occurred at the beginning of the process, we performed simulations with $N=32$ for different pore lengths. The results are given in Fig. 11a as a function of the normalized time $\langle t\rangle /\langle \tau \rangle$. The curves are plotted by using the “line-point” representation where each data point gives the required time to reach the given integer *m* state.

The stalling time is found to increase with $\ell _{\mathrm{p}}$ at $\phi _0=\phi _{0,1}$. If we look carefully, we can see that the plateau constitutes exactly $m_{\mathrm{p}}$ data points. Similar behavior is also observed for $\phi _0 < \phi _{0,1}$, for example, for the extreme case $\phi _0=\phi _{0,\infty }=0.0$ plotted at the top of the panel. The plateaus of the curves can be removed by shifting the time and the monomer number to be $\langle t\rangle -\tau _{\mathrm{n}}$ and $m - m_{\mathrm{n}}$, respectively, with the critical nucleus size $m_{\mathrm{n}}=m_{\mathrm{p}}$ and the nucleation time $\tau _{\mathrm{n}}=\langle \tau _{\mathrm{ent}}\rangle$. The resulting curves exhibit astonishing overlaps on the normalized coordinates and become independent of $\ell _{\mathrm{p}}$, as given in Fig. 11b. It shows that the entering stage and the main ejection stage are decoupled. Each stage possesses its own scaling and can be studied independently. Please notice that at $\phi _0=\phi _{0,0}\equiv 0.3$, no plateau region appears on the curve. It is because the initial volume fraction of monomers in the cavity is about equal to the volume fraction $\phi _{\mathrm{p}}$ for a monomer to be presented in the channel^39,40^. Here $\phi _{\mathrm{p}}$ can be evaluated by $\frac{1}{6}\pi \sigma ^3/(\pi r_{\mathrm{p}}^2 \sigma )\simeq 0.296$ with $r_{\mathrm{p}}=0.75\sigma$ being the pore radius. In this case, we have set both $m_{\mathrm{n}}$ and $\tau _{\mathrm{n}}$ to zero.

**Figure 12:**
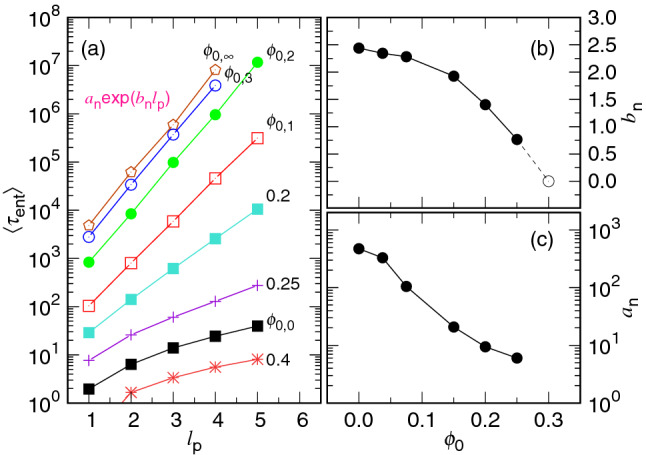
(**a**) $\langle \tau _{\mathrm{ent}}\rangle$ as a function of $\ell _{\mathrm{p}}$ in the semi-log plot. The value of $\phi _0$ is given near the corresponding curve. The data are fit by non-linear regression method via the form $a_{\mathrm{n}}\exp (b_{\mathrm{n}}\ell _{\mathrm{p}})$ for $\phi _0>\phi _{\mathrm{p}}$. (**b**) $b_{\mathrm{n}}$ vs. $\phi _0$. (**c**) $a_{\mathrm{n}}$ vs. $\phi _0$.

Figure 12a shows how the entering time varies with the pore length. In addition to $\phi _{0,g}=0.3\times 2^{-g}$ with $g=0$, 1, 2, 3, $\infty$, extra simulations have been performed at $\phi _0=0.2$, 0.25 and 0.4 to study the variation in details.

We observe that the $\langle \tau _{\mathrm{ent}}\rangle$ data falls on a straight line in the semi-log plot when $\phi _0$ is smaller than $\phi _{\mathrm{p}}\simeq 0.296$. It suggests that $\langle \tau _{\mathrm{ent}}\rangle \simeq a_{\mathrm{n}}\exp (b_{\mathrm{n}}\ell _{\mathrm{p}})$ with $a_{\mathrm{n}}$ and $b_{\mathrm{n}}$ being two parameters depending on $\phi _0$. For $\phi _0 \ge \phi _{\mathrm{p}}$, the curve bends down in the semi-log plot and does not follow well the exponential growth. $b_{\mathrm{n}}$ and $a_{\mathrm{n}}$ can be obtained by nonlinear regression fits, and the results are presented in Panels (b) and (c). The value of $b_{\mathrm{n}}$ is about 2.5 at $\phi _0=0$ and decreases with increasing $\phi _0$. The extension of the $b_{\mathrm{n}}$ curve hits zero at $\phi _0$ around 0.3, which corresponds to the $\phi _{\mathrm{p}}$ value. $a_{\mathrm{n}}$ decreases also with $\phi _0$. Different to $b_{\mathrm{n}}$, the magnitude reduces several orders.

The exponential dependence of $\langle \tau _{\mathrm{ent}}\rangle$ on $\ell _{\mathrm{p}}$ suggests an Arrhenius type of transition for the heading monomers to go across the pore. An amount of energy $E_{\mathrm{a}}=m_{\mathrm{p}}\Delta \mu _{\mathrm{cp}}$ is required for the chain to gain from the thermal fluctuations to be able to send the first monomer to the semi-space for the following process. Here $m_{\mathrm{p}}=\ell _{\mathrm{p}}/\sigma$ is the number of monomers needed to span the pore and $\Delta \mu _{\mathrm{cp}}=\mu _{\mathrm{p}}-\mu _{\mathrm{c}}$ is the chemical potential difference between the cavity and the pore, which is directly related to the volume fraction difference between the two regions. $E_{\mathrm{a}}$ is thus the “activation energy”, and the Arrhenius equation predicts the transition rate $k \propto \exp (-\frac{E_{\mathrm{a}}}{k_{{\mathrm{B}}}T})$ which gives the required passage time across the pore to be about $\exp (\frac{\Delta \mu _{\mathrm{cp}}}{\sigma k_{{\mathrm{B}}}T}\ell _{\mathrm{p}})$. A detailed calculation from the Fokker–Planck equation yields the same result: $\langle \tau _{\mathrm{ent}}\rangle \sim \eta \exp \left( \frac{m_{\mathrm{p}}\Delta \mu _{\mathrm{cp}}}{k_{{\mathrm{B}}}T}\right)$, valid for $m_{\mathrm{p}}\Delta \mu _{\mathrm{cp}}\gg k_{{\mathrm{B}}}T$ (cf. Eq. S3 in Appendix A in SI). It is the “first passage time” of the Kramers’ escape problem. We also call it the nucleation time here because the concept of nucleation has be adopted in the discussion.

If $m_{\mathrm{p}}\Delta \mu _{\mathrm{cp}}$ is not much greater than $k_{{\mathrm{B}}}T$, Eq. (S3) predicts $\langle \tau _{\mathrm{ent}}\rangle \sim \frac{\eta \ell _{\mathrm{p}}^2}{2k_{{\mathrm{B}}}T}$ for $m_{\mathrm{p}}|\Delta \mu _{\mathrm{cp}}|\ll k_{{\mathrm{B}}}T$, happened when $\phi _0$ is close $\phi _{\mathrm{p}}$, and $\langle \tau _{\mathrm{ent}}\rangle \sim (\frac{\eta \sigma }{|\Delta \mu _{\mathrm{cp}}|}) \ell _{\mathrm{p}}$ for $m_{\mathrm{p}}\Delta \mu _{\mathrm{cp}}< -k_{{\mathrm{B}}}T$, happened when $\phi _0\gg \phi _{\mathrm{p}}$. The previous situation predicts a diffusion-like motion for the head monomer to come across the pore and therefore, $\langle \tau _{\mathrm{ent}}\rangle$ is proportional to $\ell _{\mathrm{p}}^2$. The latter depicts a constant motion where the heading monomers are driven through the pore by a driving force $\frac{|\Delta \mu _{\mathrm{cp}}|}{\sigma }$, which produces a mean drift speed $\frac{|\Delta \mu _{\mathrm{cp}}|}{\eta \sigma }$. Consequently, $\langle \tau _{\mathrm{ent}}\rangle$ is proportional to $\ell _{\mathrm{p}}$. The data for $\phi _0=0.3$ and 0.4 in Panel (a) correspond to the two situations and hence, do not lie on a straight line in the semi-log plot.

**Figure 13:**
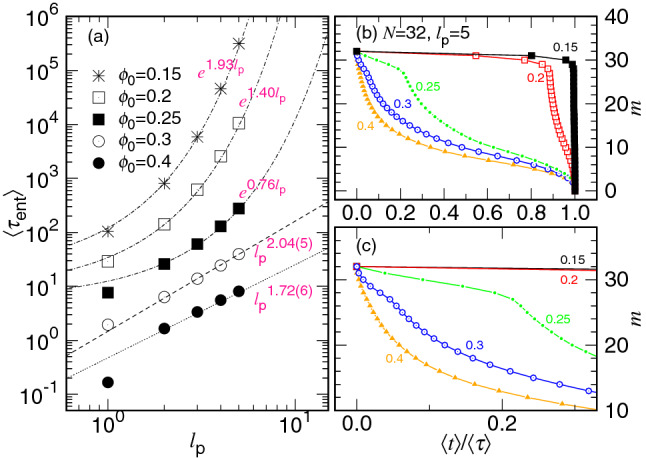
(**a**) $\langle \tau _{\mathrm{ent}}\rangle$ as a function of $\ell _{\mathrm{p}}$ in log-log scales. The chain length is 32 and the value of $\phi _0$ is indicated in the legend. (**b**) Descending of *m* plotted in the normalized time coordinate $\langle t\rangle /\langle \tau \rangle$. The pore length $\ell _{\mathrm{p}}$ is 5.0. The $\phi _0$ value is given near the corresponding curve. Panel (**c**) is a zoom-in of the panel (**b**) near the starting point $m=32$.

To make evident the scaling, we replot $\langle \tau _{\mathrm{ent}}\rangle$ against $\ell _{\mathrm{p}}$ in Fig. 13a using the log–log scales.

We can see that $\langle \tau _{\mathrm{ent}}\rangle$ behaves as $\ell _{\mathrm{p}}^{2.04(5)}$ at $\phi _0=0.3$. The $\phi _0$ value is about $\phi _{\mathrm{p}}$ and therefore corresponds to the case showing the diffusive kinetics. Increasing $\phi _0$ to 0.4 gives $\langle \tau _{\mathrm{ent}}\rangle \sim \ell _{\mathrm{p}}^{1.72(6)}$. It exhibits a super-diffusion behavior with the diffusion exponent equal to $2/1.72=1.16$. The $\phi _0$ value is not high enough to show pure constant motion in the entering stage with $\langle \tau _{\mathrm{ent}}\rangle \propto \ell _{\mathrm{p}}$. We cannot go further to investigate higher-$\phi _0$ cases, because the volume fraction has been close to the close pack value in our quasi-2D confining model. It is known that the packing fraction of a close pack in a pure two-dimensional space is $\phi _{\mathrm{cl}}^{\mathrm{(2D)}}=\frac{\pi r^2/2}{\sqrt{3}r^2}=0.9069$. In our model, the volume fraction is related to the corresponding 2D packing by $\phi _0=\phi _0^{\mathrm{(2D)}}\times \frac{2\sigma }{3H}$. A close-pack 2D arrangement thus gives $\phi _0 \simeq 0.4030$ because $H=1.5\sigma$ in this study. The value $\phi _0=0.4$ has been about the maximum volume fraction that we can investigate. For the cases with $\phi _0$ smaller than 0.3, the data follow the exponential growth with $\ell _{\mathrm{p}}$, as shown in the figure.

Figure 13b compares the descending behavior of *m* for $\ell _{\mathrm{p}}=5$ at various $\phi _0$ values. Figure 13c is the zoom-in of the curves near the starting point, $m=32$. We can see that the descending behavior, and thus the kinetics, changes when *m* goes across the number $N-m_{\mathrm{p}}=27$. For $\phi _0<\phi _{\mathrm{p}}$, the descending curve is concave in the entering stage. It changes to show convexness when $\phi _0$ becomes close or larger than $\phi _{\mathrm{p}}$.

### Processing time analysis

The scaling behavior of the spending time in an ejection process is analyzed in this section. The process can be divided into the three stages: (1) the entering stage, for the head monomer to enter and pass through the pore channel, (2) the main ejection stage, for the body of the chain to be transported from the disklike cavity, through the channel, to the semi-space, and (3) the leaving stage, for the tail monomer to leave the pore channel to the semi-space. The total processing time is thus the sum of the duration in the three stages, $\langle \tau \rangle =\langle \tau _{\mathrm{ent}}\rangle + \langle \tau _{\mathrm{ej}}\rangle + \langle \tau _{\mathrm{leav}}\rangle$. The main ejection stage can be further separated into the two stages: the confined stage, in which the chain segments suffer from the confinement of the cavity and are pressed out of it, and the non-confined stage, occurred when the pervaded space of the rested chain segments becomes smaller than the cavity size. The ejection time is thus equal to $\langle \tau _{\mathrm{ej}}\rangle = \langle \tau _{1}\rangle + \langle \tau _{2}\rangle$, the sum of the time in the two stages. We have argued that $\langle \tau _{2}\rangle$ for the non-confined stage can be furthermore split into the two terms, $\langle \tau _{\mathrm{2E}}\rangle$ and $\langle \tau _{\mathrm{2P}}\rangle$, according to the way of the chain being driven. $\langle \tau _{\mathrm{2E}}\rangle$ is the thermal escape time occurred when the chain length in the cavity is still longer than the one in the semi-space in the non-confined stage, while $\langle \tau _{\mathrm{2P}}\rangle$ is the time spent for the chain driven by the entropic pulling from the outside when the outer chain becomes longer than the remaining one in the cavity. A sketch for the free energy change in these stages has been given in Fig. 1.

**Figure 14:**
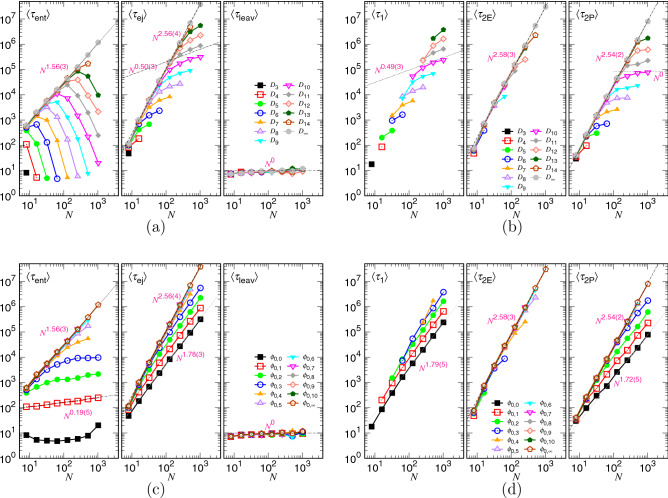
(**a**) $\langle \tau _{\mathrm{ent}}\rangle$, $\langle \tau _{\mathrm{ej}}\rangle$, $\langle \tau _{\mathrm{leav}}\rangle$, and (**b**) $\langle \tau _1\rangle$, $\langle \tau _{\mathrm{2E}}\rangle$, $\langle \tau _{\mathrm{2P}}\rangle$ as a function of *N*, plotted under the *D*-fixed condition. The value of *D* can be read in the legends, calculated by Eq. (7). (**c**) $\langle \tau _{\mathrm{ent}}\rangle$, $\langle \tau _{\mathrm{ej}}\rangle$, $\langle \tau _{\mathrm{leav}}\rangle$, and (**d**) $\langle \tau _1\rangle$, $\langle \tau _{\mathrm{2E}}\rangle$, $\langle \tau _{\mathrm{2P}}\rangle$ as a function of *N*, plotted under the $\phi _0$-fixed condition. The value of $\phi _0$ can be read in the legends, calculated by $\phi _{0,j}=0.3\times 2^{-j}$.

We first study the variations of the decomposed time as a function of *N*, in Fig. 14, under the fixing *D* and fixing $\phi _0$ conditions. Panel (a) reveals that at a given *D*, $\langle \tau _{\mathrm{ent}}\rangle$ grows like $N^{1.56(3)}$ and turns to show fast decreasing behavior as *N* becomes larger than the critical $N_*\sim (D/\sigma )^{1/\nu }$. If it is $\phi _0$ being fixed (see Panel (c)), $\langle \tau _{\mathrm{ent}}\rangle$ exhibits a change of the scaling behavior from $N^{0.19(5)}$ to $N^{1.56(3)}$ with decreasing the $\phi _0$ value. Recall that $\langle \tau _{\mathrm{ent}}\rangle$ is the nucleation time and varies as $\eta \exp \left( \frac{\ell _{\mathrm{p}}\Delta \mu _{\mathrm{cp}}}{\sigma k_{{\mathrm{B}}}T}\right)$. The fixed-$\phi _0$ study implies that $\Delta \mu _{\mathrm{cp}}$ is fixed because the chemical potential difference is directly related to the volume fraction difference between the cavity and the pore. It is thus the change of the effective friction coefficient $\eta$ which leads to the change of the scaling. In the previous three-dimensional study^39,40^, we have obtained the nucleation time $N^{0.32(2)}$ at large $\phi _0$ and $N^{1.58(6)}$ at small $\phi _0$. At that study, we have proposed a scaling argument for the change of $\eta$ from $N^{x_1}$ to $N^{1+\nu }$ with decreasing $\phi _0$. It looks that the proposed scaling fails to be extended for the two-dimensional case. The exponent larger than one for a process started by a non-confined stage, $\phi _0<\phi _*$, is understandable. Because the chain is not restricted, the entering of the head monomer across the pore needs to overcome the drag of the entire chain and also the conformational fluctuations of the chain in the cis region. Long entering time is expected with the scaling exponent of *N* larger than one, which is attributed to the contribution of the friction coefficient. If the chain is initially confined (*ie.*, $\phi _0>\phi _*$), the cavity wall bounces the chain back into the interior and the chain fluctuation is restricted. The trials for the escape of the head monomer become much easier. Consequently, the required time is shortened with a chain length exponent smaller than one, reflecting effectively on the friction coefficient. The mechanism which influences the effective friction in this entering (nucleation) stage deserves detailed investigation in the future.

The ejection time $\langle \tau _{\mathrm{ej}}\rangle$ changes from $N^{2.56(4)}$ to $N^{0.50(3)}$ with increasing *N* in Panel (a). The results are very close to the prediction given in Table 1: $\tau _{\mathrm{ej}}$ is dominated by $\tau _{2}$, scaling like $N^{1+z_{\mathrm{2P}}}$ when $N<N_*$, and by $\tau _{1}$, scaling like $N^{x_1}N_*^2$ when *N* is much larger than $N_*$, where the expected $z_{\mathrm{2P}}$ and $x_1$ exponents are $2\nu$ and 1/*d*, respectively, with $\nu =\frac{3}{d+2}=0.75$ in the two-dimensional ($d=2$) space. If the $\phi _0$-fixed condition is applied, the scaling is switched from $N^{1.78(3)}$ to $N^{2.56(4)}$ by reducing the $\phi _0$ value, as seen in Panel (c). The two exponents agree with the predictions $x_1+\frac{2}{d\nu }\simeq 1.83$ and $1+z_{\mathrm{2P}}=2.5$, respectively. Here the additional term $\frac{2}{d\nu }$ comes into the first exponent because the dominated time $\tau _1$ has the extra scaling dependence $N_*^2\sim (N/\phi _0)^{2/(d\nu )}$ on *N* as $\phi _0$ is fixed.

Panels (a) and (c) also show that the leaving time $\langle \tau _{\mathrm{leav}}\rangle$ is negligibly small in comparison with $\langle \tau _{\mathrm{ent}}\rangle$ and $\langle \tau _{\mathrm{ej}}\rangle$, and is basically a constant.

The decomposition of $\langle \tau _{\mathrm{ej}}\rangle$ shows that $\langle \tau _1\rangle$ scales like $N^{0.49(3)}$ in Panel (b) and $N^{1.79(5)}$ in Panel (d). They are close the predicted scaling $N^{x_1}$ and $N^{x_1+(2/d\nu )}$ for the *D*-fixed and $\phi _0$-fixed cases. The $\langle \tau _{\mathrm{2E}}\rangle$ data in the two panels lie basically on a line $N^{2.58(3)}$. It has been shown in Fig. 6 that $z_{\mathrm{2E}}$ is about 1.18, which gives $y_{\mathrm{2E}}\simeq 0.18$ smaller than $x_1$. The dominated scaling for $\langle \tau _{\mathrm{2E}}\rangle$ is thus $N^{2+x_1}$ according to Table 1. The $\langle \tau _{\mathrm{2P}}\rangle$ time is expected to show $N_*^{1+z_{\mathrm{2P}}}$ for $N>2N_*$ and $N^{1+z_{\mathrm{2P}}}$ for $N<2N_*$. Again, the obtained exponents, 1.72(5) at the large $\phi _0$ and 2.54(2) at the small $\phi _0$ in Panel (d), are quite close to the theoretical values $\frac{1+z_{\mathrm{2P}}}{d\nu }\simeq 1.67$ and $1+z_{\mathrm{2P}}=2.5$, respectively.

**Figure 15:**
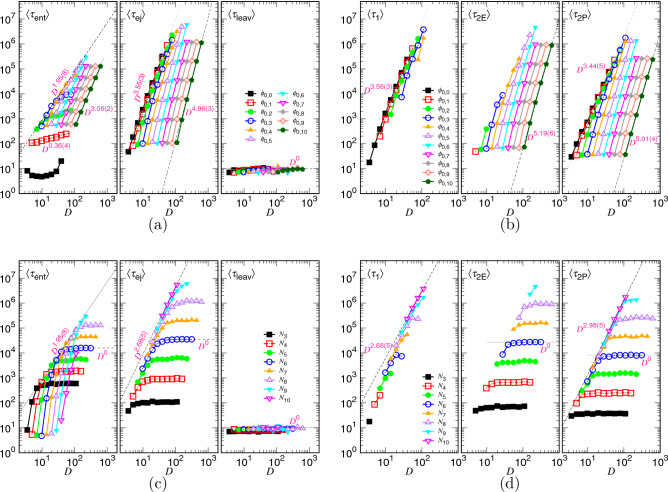
(**a**) $\langle \tau _{\mathrm{ent}}\rangle$, $\langle \tau _{\mathrm{ej}}\rangle$, $\langle \tau _{\mathrm{leav}}\rangle$, and (**b**) $\langle \tau _1\rangle$, $\langle \tau _{\mathrm{2E}}\rangle$, $\langle \tau _{\mathrm{2P}}\rangle$ as a function of *D*, plotted under the $\phi _0$-fixed condition. The value of $\phi _0$ can be read in the legends, calculated by $\phi _{0,j}=0.3\times 2^{-j}$. (c) $\langle \tau _{\mathrm{ent}}\rangle$, $\langle \tau _{\mathrm{ej}}\rangle$, $\langle \tau _{\mathrm{leav}}\rangle$, and (d) $\langle \tau _1\rangle$, $\langle \tau _{\mathrm{2E}}\rangle$, $\langle \tau _{\mathrm{2P}}\rangle$ as a function of *D*, plotted under the *N*-fixed condition. The chain length *N* can be read in the legends where $N_i=2^{i}$.

The variations of the different time components versus *D* for the $\phi _0$-fixed and *N*-fixed conditions are given in Fig. 15. We see in Panel (a) that $\langle \tau _{\mathrm{ent}}\rangle$ scales as $D^{0.36(4)}$ at $\phi _0=\phi _{0,1}\equiv 0.15$. Decreasing $\phi _0$ moves upward the curve in a parallel manner. The curve is then “reflected” toward right after meeting the demarcating line $D^{1.95(8)}$, and the scaling changes to $D^{3.06(2)}$. The results are consistent with the ones in Fig. 14c because $\phi _0\sim N\left( \frac{\sigma }{D}\right) ^2$ which yields the scaling exponent for *D* about twice of the one for *N* at a given $\phi _0$ value. Similarly, the ejection time $\langle \tau _{\mathrm{ej}}\rangle$ scales like $D^{3.55(3)}$ at large $\phi _0$ and $D^{4.96(3)}$ at small $\phi _0$. The two exponents are about twice of the ones found in Fig. 14c. The leaving time $\langle \tau _{\mathrm{leav}}\rangle$ stays small and is about constant with varying *D*.

The components of the ejection time in Panel (b) show the following scaling behaviors: $\langle \tau _1\rangle \sim D^{3.58(3)}$ (happened when $\phi _0$ is large), $\langle \tau _{\mathrm{2E}}\rangle \sim D^{5.19(8)}$ (happened when $\phi _0$ is small), and $\langle \tau _{\mathrm{2P}}\rangle \sim D^{3.44(5)}$ at large $\phi _0$ and $D^{5.01(4)}$ at small $\phi _0$. The exponents are found to be the double of the ones in Fig. 14d.

Figure 15c reveals that $\langle \tau _{\mathrm{ent}}\rangle$ grows faster than a power law when *D* is smaller than the critical value $D_*\sim \sigma N^{\nu }$ and becomes horizontal when $D>D_*$, if *N* is fixed. The scaling line $D^{1.95(8)}$ in the figure tells how $\langle \tau _{\mathrm{ent}}\rangle$ varies at the critical diameter. The ejection time $\langle \tau _{\mathrm{ej}}\rangle$ grows initial as $D^{2.68(5)}$, following the predicted behavior $D^{2/\nu }$, and turns to becomes horizontal as $D>D_*$. Panel (d) shows that the component $\langle \tau _1\rangle$ exhibits a $D^{2.68(5)}$ scaling. $\langle \tau _{2E}\rangle$ is about constant in the concerned large *D* region and $\langle \tau _{2P}\rangle$ possesses the exponent value 2.98(5) for $D<D_*$ and turns to be leveled-off for $D>D_*$. The previous exponent is close to the theoretical value $\frac{1+z_{\mathrm{2P}}}{\nu }$.

**Figure 16:**
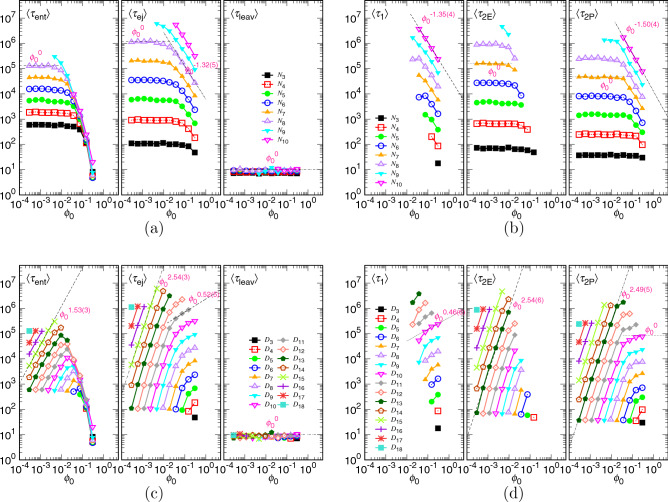
(**a**) $\langle \tau _{\mathrm{ent}}\rangle$, $\langle \tau _{\mathrm{ej}}\rangle$, $\langle \tau _{\mathrm{leav}}\rangle$, and (**b**) $\langle \tau _1\rangle$, $\langle \tau _{\mathrm{2E}}\rangle$, $\langle \tau _{\mathrm{2P}}\rangle$ as a function of $\phi _0$, plotted under the *N*-fixed condition. The chain length *N* is indicated in the legends with $N_i=2^i$. (**c**) $\langle \tau _{\mathrm{ent}}\rangle$, $\langle \tau _{\mathrm{ej}}\rangle$, $\langle \tau _{\mathrm{leav}}\rangle$, and (**d**) $\langle \tau _1\rangle$, $\langle \tau _{\mathrm{2E}}\rangle$, $\langle \tau _{\mathrm{2P}}\rangle$ as a function of $\phi _0$, plotted under the *D*-fixed condition. The *D* value can be found in the legends, calculated by Eq. (7).

Figure 16 presents the variations of the different time components against the initial volume fraction $\phi _0$. With *N* being fixed, Panels (a) and (b) are, in fact, the replots of Fig. 15c,d through the abscissa mapping $\phi _0 \simeq N\left( \sigma /D\right) ^{2}$. In the plots, $\langle \tau _{\mathrm{ent}}\rangle$ is flat in the small $\phi _0$ region and decreases quickly as $\phi _0$ passes a critical value $\phi _{0*}$. The curves join a master dropping curve and become zero (minus infinity in the log scale) at $\phi _0 \simeq \phi _{\mathrm{p}}$. $\langle \tau _{\mathrm{ej}}\rangle$ shows a power-law decreasing behavior $\phi _0^{-1.32(5)}$ as $\phi _0>\phi _{0*}$. In the confined stage, we observe the scaling $\langle \tau _{1}\rangle \sim \phi _0^{-1.35(4)}$. In the non-confined stage, $\langle \tau _{\mathrm{2E}}\rangle$ is about constant while $\langle \tau _{\mathrm{2P}}\rangle$ changes the scaling from $N^{1+z_{\mathrm{2P}}}$ (independent of $\phi _0$) to be $N_*^{1+z_{\mathrm{2P}}}\sim \left( N/\phi _0\right) ^{(1+z_{\mathrm{2P}})/(d\nu )}$ when passing the critical point. The obtained exponent $-1.50(4)$ for $\phi _0$ is somewhat larger than the predicted value $-\frac{1+z_{\mathrm{2P}}}{2\nu }\simeq -1.67$.

The plots in Fig. 16c,d can be related to Fig. 14a,b via the same mapping $\phi _0 \simeq N(\sigma /D)^2$ but it is the *D* value being fixed. Therefore, the scaling exponents extracted from the two sets of figures should be identical. We can see that $\langle \tau _{\mathrm{ent}}\rangle$ increases with $\phi _0$ with a consistent exponent 1.53(3) for $\phi _0<\phi _{0*}$. In the region $\phi _0>\phi _{0*}$, the decreasing curves collapse and tend to be zero at $\phi _0$ around 0.3. $\langle \tau _{\mathrm{ej}}\rangle$ shows $\phi _0^{2.54(3)}$ for the cases experiencing only the non-confined stage, while it changes to $\phi _0^{0.52(5)}$ for the ones experiencing the confined and then non-confined stages. The scaling exponents for $\langle \tau _{1}\rangle$ and $\langle \tau _{\mathrm{2E}}\rangle$ are 0.46(6) and 2.54(6), respectively. The $\langle \tau _{\mathrm{2P}}\rangle$ data show consistent scaling $\phi _0^{2.49(5)}$ and tend to be leveled off as $\phi _0$ is large.

**Figure 17:**
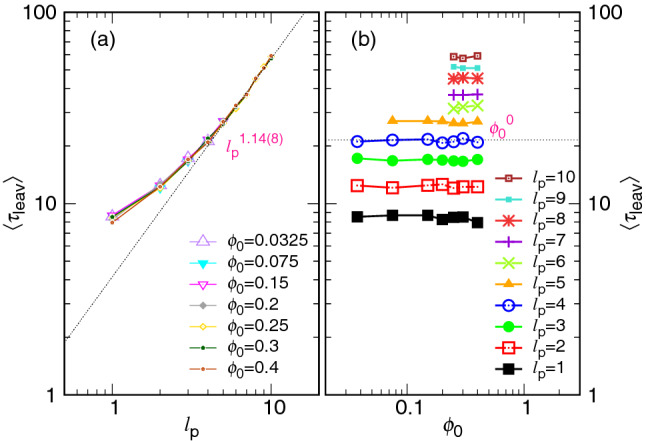
(**a**) $\langle \tau _{\mathrm{leav}}\rangle$ as a function of $\ell _{\mathrm{p}}$ at various $\phi _0$ values, indicated in the legend. (**b**) $\langle \tau _{\mathrm{leav}}\rangle$ vs. $\phi _0$ with $\ell _{\mathrm{p}}$ being fixed. The chain length is 32.

As seen in the above figures, the leaving time $\langle \tau _{\mathrm{leav}}\rangle$ does not change basically with varying the *N*, *D*, and $\phi _0$ variables. It is because the relevant quantity, the pore length $\ell _{\mathrm{p}}$, is fixed in the study. To explore the behavior of $\langle \tau _{\mathrm{leav}}\rangle$, we have performed simulations for $N=32$ by varying $\ell _{\mathrm{p}}$. The results are shown in Fig. 17.

We can see in Panel (a) that the leaving time increases with the pore length at various $\phi _0$ values and tends to grow as $\ell _{\mathrm{p}}^{1.14(8)}$. Panel (b) reveals that the $\langle \tau _{\mathrm{leav}}\rangle$ value is basically independent of the initial volume fraction $\phi _0$. The reason for the properties is explained below. At the final moment of an ejection process (the leaving stage), the chain tail is driven across the pore channel by the chemical potential difference $\Delta \mu _{\mathrm{ps}}$ between the pore and the outer semi-space. The initial volume fraction $\phi _0$ thus has no effect on the leaving mechanism. The leaving time is predicted to be about $\Delta {\mathfrak {t}}_0\left( \frac{k_{{\mathrm{B}}}T}{|\Delta \mu _{\mathrm{ps}}|\sigma }\right) \ell _{\mathrm{p}}$, which is proportional to the pore length. What we have observed agrees mainly with the prediction.

## Discussions and conclusions

**Figure 18:**
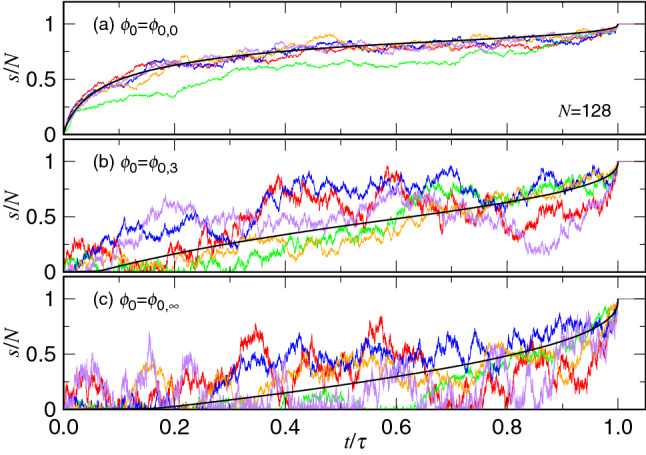
Evolution of the normalized coordinate *s*/*N* versus the normalize time $t/\tau$ in five typical ejection processes, plotted in colored curves, at (**a**) $\phi _0=\phi _{0,0}$, (**b**) $\phi _0=\phi _{0,3}$, and (**c**) $\phi _0=\phi _{0,\infty }$. The chain length is $N=128$. The mean evolution is plotted on top of the five curves in black color in each panel.

To make connection with applications, we present time variational curves of the ejection coordinate *s* in Fig. 18. Because the processing time $\tau$ of an ejection varies a lot from one process to the others, the curves are plotted by using the normalized time and ejection coordinates, $t/\tau$ and *s*/*N*, for comparison. Five typical curves, together with the mean curve averaged over 500 independent runs, are presented at the three selected initial volume fractions.

At the large volume fraction, $\phi _0=\phi _{0,0}=0.3$, the differences between individual curves is relatively small (refer to Panel (a)). The ejection follows mainly the path of the mean curve. In other words, the evolution of the ejection is quite deterministic. As $\phi _0$ decreases, the evolution becomes more and more stochastic. The random-walk nature of the process leads to large fluctuations, upward and downward in the *s*-coordinate as shown in Panel (b). At zero initial volume fraction, $\phi _0=\phi _{0,\infty }$, a large free energy barrier has to be overcome by the system. The chain struggles for leaving the cis side. We can see that in addition to the large variation, the curve bounces so many times with the bottom line $s=0$, until the chain finds a way to go out of the trap and finally reaches the trans side.

In applications, a large initial volume fraction is generally preferable so that the ejection can be effectuated in a controllable (deterministic) way. Figure 18a has revealed that an individual ejection spends the first $20\%$ of the time on ejecting about two thirds of the chain out of the cavity and the rest the remaining $80\%$ of the time to complete it. The proportion of the chain to be ejected in the first $20\%$ time is even higher if the chain length *N* becomes longer by keeping the same $\phi _0$ value. A process like this definitely gives us a sensation that the ejection is drastically slowed down or seemly “halted” after spitting out a considerable long segment from the confining cavity. It might explain the experimental observations for the inhibition of DNA ejection from bacteriophages under certain conditions^12,24,56^. In particular, increasing the osmotic pressure from the outer solution could be regarded as an effective reduction of the osmotic pressure difference between the inside and outside of the cavity. Thus, the ejection might show an evolution curve similar to the small $\phi _0$ cases given in Panels (b) and (c) of the figure.

In this study, we have generalized the ejection theory for polymer to a two-dimensional space. The kinetics equation was derived from the Onsager’s variational principle by using the free energy obtained in the scaling theory of polymer physics. The ejection speed was shown to scale as $m^{z_1}/(N^{x_1}N_*^{z_1+1})$ in the confined stage and $m^{-z_{\mathrm{2P}}}$ in the non-confined stage with $x_1=1/d$, $z_1=1/(d\nu -1)$, and $z_{\mathrm{2P}}=2\nu$, where *m* is the number of monomers in the circular cavity and $N_*$ is the critical number of monomers to distinguish the confined and the non-confined stages at a given cavity diameter *D*. The analysis showed that the non-confined stages can be further divided into two substages, determined by whether the chain length in the cavity is still longer than the one in the outer space or not. The spending time can be calculated in these stages and the ejection time $\tau _{\mathrm{ej}}$ is equal to $\tau _1+\tau _{\mathrm{2E}}+\tau _{\mathrm{2P}}$. Because the pore has finite length, non-negligible time is required for the head monomer to traverse the pore channel to be able to start ejecting chain segments into the outer space. The time spent for the tail monomer to go across the pore channel to end the process is generally short. The two events define two additional stages before and after the main ejection stage, called the entering stage and the leaving stage. In the entering stage, $\tau _{\mathrm{ent}}$ can be calculated by solving the Kramers escape problem while $\tau _{\mathrm{leav}}$ for the leaving stage is derived from the chemical potential difference. The total processing time $\tau$ is thus the sum of the three components, $\tau _{\mathrm{ent}}$, $\tau _{\mathrm{ej}}$, and $\tau _{\mathrm{leav}}$. Each has its own scaling behavior. In the entering stage, $\tau _{\mathrm{ent}}$ is described by Eq. (S3) in SI. It exhibits an exponential increase with the pore length, like $\eta \exp \left( \frac{\ell _{\mathrm{p}}\Delta \mu _{\mathrm{cp}}}{\sigma k_{{\mathrm{B}}}T}\right)$, for $\phi _0$ much smaller than $\phi _{\mathrm{p}}$, the local volume fraction of a monomer presented in the pore channel. If $\phi _0$ is around $\phi _{\mathrm{p}}$, $\tau _{\mathrm{ent}}$ is characterized by a diffusive time $\frac{\eta \ell _{\mathrm{p}}^2}{2k_{{\mathrm{B}}}T}$. In the main ejection stage, if the confined stage is the dominated contribution, $\tau _{\mathrm{ej}}$ scales as $N^{x_1+(2/d\nu )}\phi _0^{-2/d\nu }$, or equivalently $N^{(2d+7)/3d}\phi _0^{-(2d+4)/3d}$ by replacing $\nu$ with $3/(d+2)$. If the dominating is the non-confined stage, the scaling of $\tau _{\mathrm{ej}}$ is a combination of the two terms, $N^{2+x_1}$ and $N^{1+z_{\mathrm{2P}}}$, which can be expressed as a function of *d* as $N^{2+(1/d)}$ and $N^{(d+8)/(d+2)}$, respectively. In the leaving stage, the spending time is $\tau _{\mathrm{leav}}\sim \ell _{\mathrm{p}}\left( \frac{k_{{\mathrm{B}}}T}{\sigma |\Delta \mu _{\mathrm{ps}}|}\right)$.

Elaborated molecular dynamics simulations have been performed to verify the generalized ejection theory in the two-dimensional space. By varying the chain length *N*, the cavity diameter *D*, and thus the initial volume fraction $\phi _0$, we demonstrated that the ejection speed can be well described by the kinetics equation Eq. (11) with the scaling exponents in consistent with the predictions (Figs. 3, 4, 5, 6, 7). The study for the local minimum of the ejection speed did show that the critical number $N_*$ varies as $D^{1/\nu }$ with $\nu =0.75$ being the two-dimensional Flory exponent (Fig. 8). The time evolution of *m* revealed that a nucleation-like stage occurs prior to the main ejection stage. The trimmed curves were described by Eqs. (12) and (13), respectively, when *m* approaches the starting and the ending point of the ejection stage, with the exponents $\zeta _1=1.0$ and $\zeta _2=0.4$ (Figs. 9, 10, 11). The entering time was then examined by varying the pore length, and shown to fulfill the properties of the first passage time obtained from the Kramers escape problem (Figs. 12, 13). The total processing time was decomposed in Figs. 14, 15, 16 and the scaling behaviors were verified carefully in each stage by varying *N*, *D*, $\phi _0$. The results support the predicted behaviors given in Table 1. Finally, the leaving time was checked and demonstrated linearly proportional to the pore length (Fig. 17). Owing to the consistency of the various scaling behaviors in the different stages, we conclude that the free energy landscape of ejection should be as sketched as in Fig. 1.

The ejection theory has been demonstrated valid both in this two-dimensional study and in the previous three-dimensional investigation^39,40^. It is a very robust theory able to predict variations of various quantities such as the ejection speed, the ejection coordinate, and the ejection time. A process starting with a high density as well as with zero volume fraction can be well explained under the same theoretical framework. In other words, the theory can be used to understand the phenomena of polymer ejection and the polymer translocation across a wall. It shows how complicated the kinetics can be in releasing a confined or non-confined polymer through a channel. It allows people to get insight of the ejection physics and helps researchers in the development of various ejection devices for two-dimensional or three-dimensional applications.

## Supplementary Information

Supplementary Information 1
