# Supplementary material for: A general theory of polymer ejection tested in a quasi two-dimensional space
Source: Sci Rep. 2021 Jul 19;11:14721. doi: 10.1038/s41598-021-94054-2 (PMC8289874; doi:10.1038/s41598-021-94054-2)
Supplement: Supplementary file 1 — Supplementary Information 1 [file 41598_2021_94054_MOESM1_ESM.pdf]

Supplementary Information
A General Theory of Polymer Ejection Tested in a Quasi Two-Dimensional Space

Pai-Yi Hsiao[1,2,*] and Wei-Yei Chen[1]
[1] Department of Engineering and System Science, National Tsing Hua University, Taiwan, R.O.C.
[2] Institute of Nuclear Engineering and Science, National Tsing Hua University, Taiwan, R.O.C.
[*] Corresponding author, email: pyhsiao@ess.nthu.edu.tw; pyhsiao@mx.nthu.edu.tw

APPENDIX A

Consider the following Fokker-Planck equation for the conditional probability density $P(x,t|x_0,t_0)$ of finding a particle at a reaction coordinate x at time t, given that the particle is initially at the coordinate x_0 at time t_0:

$$\frac{\partial}{\partial t}P(x,t|x_0,t_0) = -\frac{\partial}{\partial x}\left(V(x)P(x,t|x_0,t_0)\right) + \frac{\partial^2}{\partial x^2}\left(D(x)P(x,t|x_0,t_0)\right) \tag{S1}$$

In the equation, $V(x) = f(x)/\eta(x)$ is the drift velocity and $D(x) = k_\mathrm{B}T/\eta(x)$ is the diffusion coefficient where $\eta(x)$ is a coordinate-dependent friction coefficient and $f(x)$ is the thermodynamic force which is equal to the negative gradient of the free energy $F(x)$. The mean first passage time for a particle to escape an interval region $[a,b]$ from a point x inside it can be shown to be

$$\mathcal{T}(x;a,b) = \int_x^b dy\, e^{F(y)/k_\mathrm{B}T} \int_a^y dz\, \frac{\eta(z)}{k_\mathrm{B}T} e^{-F(z)/k_\mathrm{B}T} \tag{S2}$$

where a reflecting boundary is set at a and an absorbing boundary at b [1].

Let the reaction coordinate be the state variable s, and set $a = -m_\mathrm{p}$ and $b = N$ for our study. In the entering stage, the free energy landscape is $F(s) = (s+m_\mathrm{p})\Delta\mu_\mathrm{cp}$, as shown in Figure 1 in the main paper. The required time to pass the stage can be calculated by $\mathcal{T}(-m_\mathrm{p};-m_\mathrm{p},N) - \mathcal{T}(0;-m_\mathrm{p},N)$, which gives

$$\tau_\mathrm{ent} = \frac{\eta\sigma^2}{k_\mathrm{B}T}\left(\frac{k_\mathrm{B}T}{\Delta\mu_\mathrm{cp}}\right)^2 \left[\exp\left(\frac{m_\mathrm{p}\Delta\mu_\mathrm{cp}}{k_\mathrm{B}T}\right) - 1 - \frac{m_\mathrm{p}\Delta\mu_\mathrm{cp}}{k_\mathrm{B}T}\right]. \tag{S3}$$

In the non-confined stage, a second thermal escape substage can take place when $s < m < N_*$ for the chain length lying between $N_* + m_\mathrm{p}$ and $2N_* + m_\mathrm{p}$. The free energy looks similar to Curve III in Figure 1 and can be expressed by a piecewise function

$$F(s) = \begin{cases} s\Delta\mu_\mathrm{cp} + k_\mathrm{B}T\left[\left(\frac{N-m_\mathrm{p}}{N_*}\right)^{z_1+1} - 1\right] & \text{if } -m_\mathrm{p} \le s \le 0 \\ k_\mathrm{B}T\left[\left(\frac{N-m_\mathrm{p}-s}{N_*}\right)^{z_1+1} - 1\right] & \text{if } 0 \le s \le s_* \\ k_\mathrm{B}T(1-\gamma_1)\ln\frac{(s+1)(N-m_\mathrm{p}-s+1)}{(s_*+1)(N-m_\mathrm{p}-s_*+1)} & \text{if } s_* \le s \le N-m_\mathrm{p} \\ (s-N+m_\mathrm{p})\Delta\mu_\mathrm{ps} + k_\mathrm{B}T(1-\gamma_1)\ln\frac{(N-m_\mathrm{p}+1)}{(s_*+1)(N-m_\mathrm{p}-s_*+1)} & \text{if } N-m_\mathrm{p} \le s \le N \end{cases} \tag{S4}$$

Here $s_* = N - m_\mathrm{p} - N_*$ and we have set $F(s_*) = 0$. The required time τ_2E for the thermal escape substage can be calculated by $\mathcal{T}(s_*;-m_\mathrm{p},N) - \mathcal{T}(\frac{N-m_\mathrm{p}}{2};-m_\mathrm{p},N)$. Assuming that $\eta \sim \eta_0 m^{y_\mathrm{2E}}$ in the substage and $N \gg m_\mathrm{p}$, we have

$$\tau_\mathrm{2E} \sim \frac{\eta_0\sigma^2}{k_\mathrm{B}T}\left[N^{2+x_1}I_1(\tilde{s}_*) + N^{2+y_\mathrm{2E}}I_\mathrm{2E}(\tilde{s}_*)\right] \tag{S5}$$

where $\tilde{s}_* = s_*/(N-m_\mathrm{p})$ has a value lying between 0 and 1/2, and

$$I_1(\tilde{s}_*) = \int_{\tilde{s}_*}^{\frac{1}{2}} d\tilde{y} \int_0^{\tilde{s}_*} d\tilde{z}\, \left[\frac{\tilde{y}(1-\tilde{y})}{\tilde{s}_*(1-\tilde{s}_*)}\right]^{1-\gamma_1} \exp\left(1 - \left[\frac{1-\tilde{z}}{1-\tilde{s}_*}\right]^{z_1+1}\right) \tag{S6}$$

$$I_\mathrm{2E}(\tilde{s}_*) = \int_{\tilde{s}_*}^{\frac{1}{2}} d\tilde{y} \int_{\tilde{s}_*}^{\tilde{y}} d\tilde{z}\, \left[\frac{\tilde{y}(1-\tilde{y})}{\tilde{z}(1-\tilde{z})}\right]^{1-\gamma_1} (1-\tilde{z})^{y_\mathrm{2E}} \tag{S7}$$

The two dimensionless functions I_1 and $I_{2\mathrm{E}}$ can be evaluated numerically.

REFERENCES

[1] C. W. Gardiner, *Handbook of stochastic methods for physics, chemistry and the natural sciences*, 3rd ed. (Springer-Verlag, Berlin, 2004).
